# Genomics of Developmental Plasticity in Animals

**DOI:** 10.3389/fgene.2019.00720

**Published:** 2019-08-07

**Authors:** Elvira Lafuente, Patrícia Beldade

**Affiliations:** ^1^Instituto Gulbenkian de Ciência, Oeiras, Portugal; ^2^CNRS-UMR5174, Université Paul Sabatier, Toulouse, France; ^3^Centre for Ecology, Evolution, and Environmental Changes, Faculty of Sciences, University of Lisbon, Lisbon, Portugal

**Keywords:** developmental plasticity, reaction norms, environmentally responsive genes, genomics of plasticity, plasticity variation

## Abstract

Developmental plasticity refers to the property by which the same genotype produces distinct phenotypes depending on the environmental conditions under which development takes place. By allowing organisms to produce phenotypes adjusted to the conditions that adults will experience, developmental plasticity can provide the means to cope with environmental heterogeneity. Developmental plasticity can be adaptive and its evolution can be shaped by natural selection. It has also been suggested that developmental plasticity can facilitate adaptation and promote diversification. Here, we summarize current knowledge on the evolution of plasticity and on the impact of plasticity on adaptive evolution, and we identify recent advances and important open questions about the genomics of developmental plasticity in animals. We give special attention to studies using transcriptomics to identify genes whose expression changes across developmental environments and studies using genetic mapping to identify loci that contribute to variation in plasticity and can fuel its evolution.

## Adaptive Developmental Plasticity

**Figure 1:**
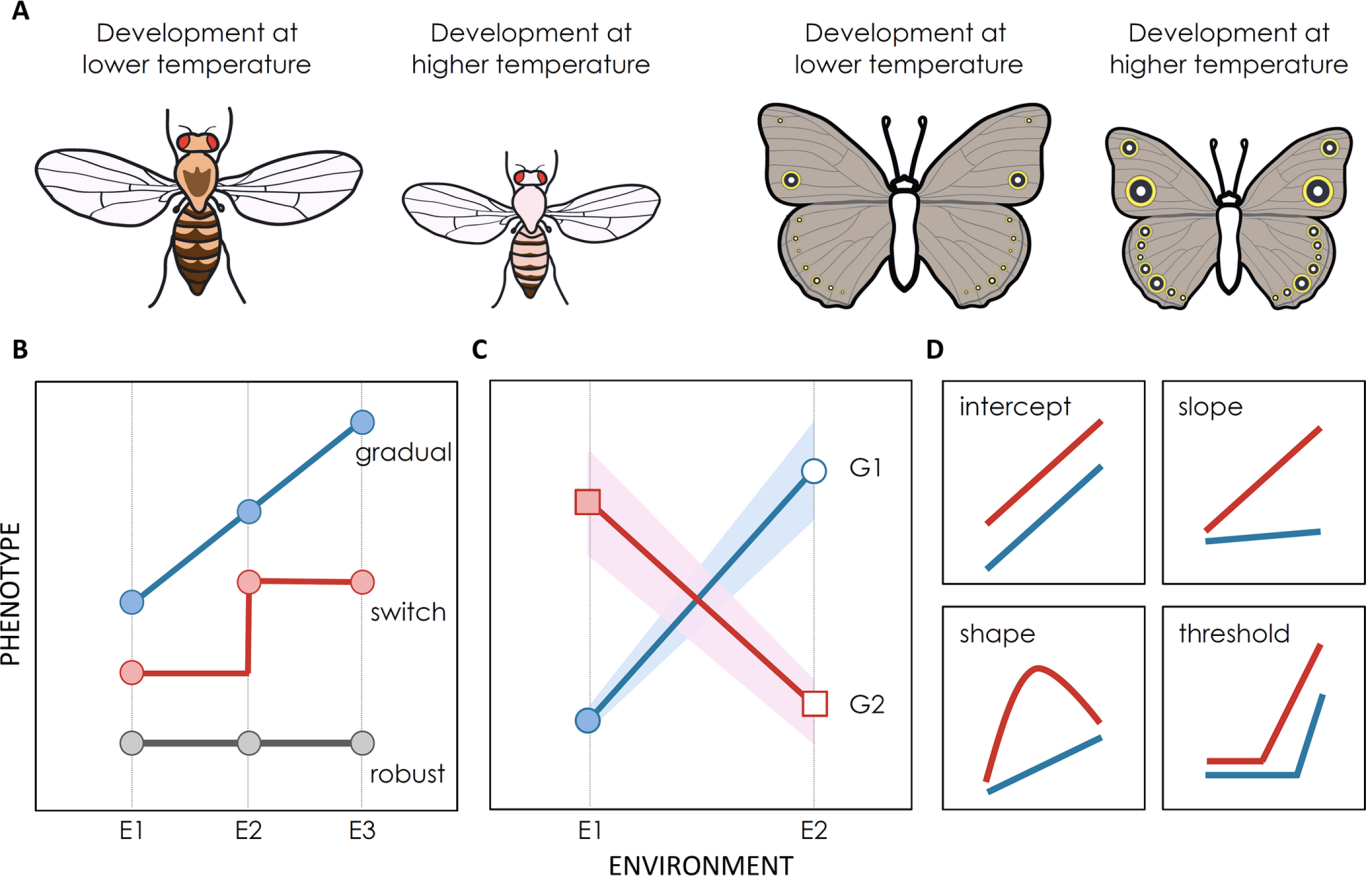
Environmental effects on phenotype expression. **(A)** Illustration of two emblematic examples of developmental plasticity: thermal plasticity in body size and pigmentation in *D. melanogaster* flies and *B. anynana* butterflies. **(B–D)** Illustration of reaction norms where phenotype is represented as a function of environmental conditions. Reaction norms can differ between traits (for the same genotype and in relation to the same cue), between environmental cues (for the same genotype and trait), and between genotypes (for the same cue and trait). **(B)** Reaction norms represent environmental effects on trait expression which can be of different general types: unresponsive phenotype robust to environmental variation (gray line), a continuous response (blue line), a switch-like relationship with discrete alternative phenotypes above and below some environmental threshold (red line). **(C)** Schematic representation of phenotypic values for two genetic backgrounds (G1 and G2) developing under two environmental conditions (E1 and E2). Total phenotypic variation in a population can be partitioned into genetic variation (difference between circles and squares), environmental variation (difference between filled and empty symbols) and GxE variation (difference between red and blue lines). There is also an intra-genotype, intra-environment component of variation, often assigned to “noise”, which is represented by the shadowing around the lines. **(D)** Genotypes can differ in distinct properties of reactions norms, such as intercept, slope, shape, and/or threshold at which the phenotype responds to environmental variation. In some cases it is possible to use genetic mapping approaches to identify the genes that contribute to such inter-genotype differences in reaction norms.

Phenotypic variation is the raw material for natural selection to drive adaptation and speciation. Studies on a variety of taxa have provided valuable insights into the molecular mechanisms that produce phenotypic variants and into the evolutionary forces and ecological conditions that shape phenotypic frequencies in populations (see Carroll, 2000; Stern, 2000; Whitehead and Crawford, 2006; Laland, 2015). We have accumulated detailed data of the genetic basis of variation for many adaptive traits (including morphology, pigmentation, behavior, and life histories) in relation to diverse selective agents (including communication, mating, and infection; e.g. Sasabe et al., 2007; Greenwood et al., 2011; Martins et al., 2014). Progress also includes studies that explore the role of environmental conditions as instructive agents that can affect the production, more than just the frequency, of phenotypic variants (Przybylo et al., 2000; Chakir et al., 2002; Gilbert and Epel, 2009; Torres-Dowdall et al., 2012; Day and McLeod, 2018; Fraimout et al., 2018; Sentis et al., 2018). This environmental regulation of phenotype expression, by which a genotype can produce different phenotypes depending on the external conditions experienced, is called phenotypic plasticity. During adulthood, environmentally induced phenotypes are often reversible, as is the case with rapid metabolic, physiological, or behavioral alterations (e.g. Oufiero and Whitlow, 2016; Abbey-Lee and Dingemanse, 2019; Guzzo et al., 2019). In the case of developmental plasticity, external environmental cues influence developmental rates and/or trajectories and lead to changes in adult phenotypes that are often irreversible (reviewed by, e.g., Nijhout, 2003a; West-Eberhard, 2003; Beldade et al., 2011; Moczek et al., 2011; Edelaar et al., 2017; Klingenberg, 2019; **Figure 1**). The effects of environmental conditions can also affect the phenotype of future generations and this type of trans-generational plasticity is receiving increased attention (e.g. Gapp et al., 2014; Walsh et al., 2016; Heckwolf et al., 2018; Schulz et al., 2019; Fuxjäger et al., 2019). This review focuses on intra-generational developmental plasticity in animals, including an overview of its reciprocal effects on evolution (**Box 1**) and an emphasis on the genomic underpinnings of its regulation and of its evolution. A number of insightful reviews have provided a historical perspective of the concept and importance of plasticity and non-genetic inheritance in the study of evolution (e.g. DeWitt and Scheiner, 2004; Pigliucci, 2007; Bossdorf et al., 2008; Mesoudi et al., 2013; Deans et al., 2015; Nicoglou, 2015; Charlesworth et al., 2017; Futuyma, 2017; Svensson, 2018).

Box 1 **Reciprocal interactions between plasticity and evolution.**

We illustrate the key aspects of the relationship between plasticity and evolution, whose reciprocal interactions are further discussed in the main text. On the one hand, plasticity is itself a heritable trait that is under selection and can evolve (blue arrow). Above the arrow, we list three of the genetic mechanisms relevant for the evolution of plasticity. On the other hand, plasticity has been proposed to impact adaptive evolution (red arrow). Below the arrow, we list three of the proposed non-mutually exclusive and partly overlapping hypotheses by which plasticity might positively impact adaptive evolution and diversification.

**Figure:**
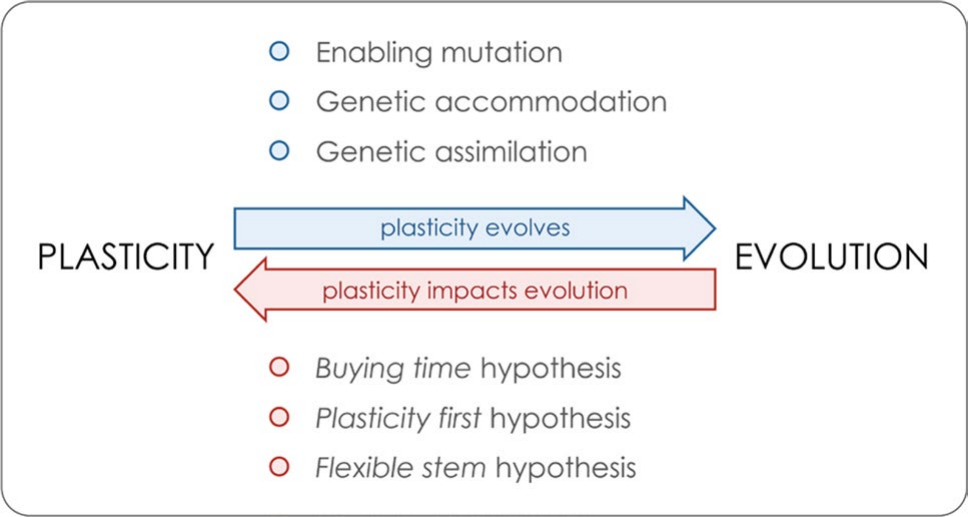


**Enabling mutation **refers to a**genetic alteration conferring environmental sensitivity to a phenotype that was originally not plastic. This****allows the expression of plasticity, which can thereafter be shaped by selection (e.g. Suzuki and Nijhout, 2006). Note that although such a mutation refers to the acquisition of plasticity from a non-plastic ancestral, it has been proposed that some level of plasticity is likely the ancestral condition for developmental processes (e.g. Newman and Müller, 2000; Nijhout, 2003a; Nijhout et al., 2017).

**Genetic accommodation **refers to the process by which selection shapes the properties and/or magnitude****of a plastic response (West-Eberhard, 2003, West-Eberhard, 2005; Crispo, 2007). Such changes in plasticity have been shown to occur under artificial and natural selection and include the evolution of both increased and decreased plasticity (e.g. Gomez-Mestre and Buchholz, 2006; Suzuki and Nijhout, 2006; Ledon-Rettig et al., 2008; Kulkarni et al., 2017).

**Genetic assimilation** refers to the process of genetic accommodation by which there is the fixation of what were previously environmentally induced phenotypes. This process is believed to have been involved in the transition from polyphenisms to polymorphisms and sustain a mechanism by which plasticity can promote phenotypic diversification (e.g. Schlichting and Wund, 2014; Ehrenreich and Pfennig, 2016; Schneider and Meyer, 2017).

The **“buying time” hypothesis** suggests that when colonizing a new habitat or facing environmental perturbation, a “plastic population” can first adjust to the new conditions by expressing distinct plastic phenotypes and thereby persist enough time for new mutations to happen and fuel adaptive evolution (e.g. Chevin and Lande, 2009; Corl et al., 2018; Pennisi, 2018). As discussed in the main text, there is a rationale and specific examples why plasticity might hurt rather than help in the face of new environmental conditions (e.g. Langerhans and Dewitt, 2002; Ghalambor et al., 2007; Oostra et al., 2018).

The **“plasticity first” hypothesis **proposes that plasticity can initiate and accelerate the rate of phenotypic change in that plastic adaptive phenotypes can emerge earlier and faster than phenotypic changes due to genetic mutation. Under this model, multiple initial alternative phenotypes generated by developmental plasticity can become genetically fixed by genetic assimilation (e.g. Levis and Pfennig, 2016; Levis and Pfennig, 2018).

The** “flexible stem” hypothesis **relies on the exact same idea but focuses explicitly on plasticity in ancestral species/populations facilitating phylogenetic diversification. This will occur in cases where plasticity produced alternative phenotypes in sister lineages that were later on fixed by genetic assimilation (West-Eberhard, 2003; Wund et al., 2008; Schneider and Meyer, 2017).

### Inductive and Selective Environments

Developmental plasticity is pervasive in nature, with many environmental factors affecting the expression of different traits in a variety of species. Emblematic examples of developmental plasticity include temperature-dependent sex determination in reptiles (Merchant-Larios and Díaz-Hernández, 2013; Mitchell et al., 2018; Noble et al., 2018), nutrition-dependent caste determination in social insects (Maleszka, 2008; Smith et al., 2008), and density-dependent production of dispersing morphs in swarming locusts (Pener and Simpson, 2009; Ernst et al., 2015) and other insects (e.g. aphids; Braendle et al., 2006).

**Figure 2:**
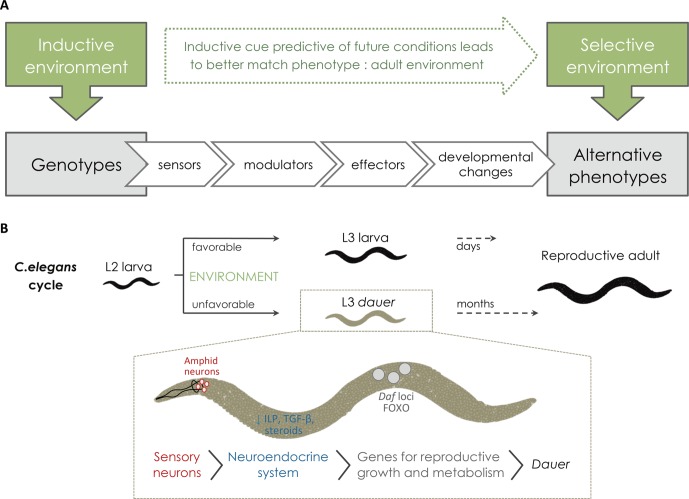
From the inductive environment to the production of alternative phenotypes. **(A)** For the external conditions to affect development environmental cues need to be perceived or sensed (*via* sensor mechanisms) and this information needs to be transmitted to the developing tissues by internal signals (*via* modulators, such as hormones). Upon receiving such signals, local changes in gene expression (effector genes) and/or function will modify development and give rise to alternative adult phenotypes. In cases of adaptive plasticity, alternative adult phenotypes are better suited to their respective environments. **(B)** Schematic representation of *C. elegans* life cycle and *dauer* formation showing examples of the molecular players involved in the plastic response (reviewed in Fielenbach and Antebi, 2008). Under favorable environments, *C. elegans* progresses rapidly through larval development until adulthood. When facing unfavorable conditions, such as high population density, starvation, and/or high temperature, *C. elegans* undergoes development to a specialized larval diapause stage called *dauer*, which can last several months. The process starts with the perception of the inductive environmental cues (e.g. ascaroside pheromones, nutrients, and/or temperature) *via* sensory organs, called amphid neurons. Amphid neurons then transduce external signals into endocrine signals (*via* G protein-coupled receptors). When hormonal signaling mechanisms (e.g. TGF-β, insulin, and steroids) are down regulated, they induce *dauer* diapause. Serotonergic signaling influences the production of TGF-β and insulin-like peptides (ILPs). Down-regulated ILP and TGF-β production results in nuclear translocation of different genes, including DAF genes and FOXO, that will then turn on genes for stress resistance, *dauer* formation, and longevity.

In the study of the regulation and evolution of developmental plasticity, it is often useful to distinguish between the environmental factor(s) that can induce changes in development, hereafter called inductive environmental cue(s), and the environmental factor(s) responsible for fitness differences between induced phenotypes, hereafter called the selective environment (**Figure 2A**). The relationship between inductive and selective environments is, in fact, of fundamental importance for the evolution of plasticity (Nijhout, 2003a). In some cases, the main inductive and selective environmental factors are the same, such as with the thermally induced changes in body size that influence the thermo-regulation of *Drosophila melanogaster* adults (Ghosh et al., 2013; **Figure 1A**). In others, the inductive cue is predictive of future environmental conditions but not the main selective agent. This is often the case with polyphenisms, which are environmentally induced alternative discrete phenotypes (see Nijhout, 2003a; Simpson et al., 2011) that are common in relation to alternating seasons (see Kivelä et al., 2013). In the polyphenism of *Bicyclus anynana* butterflies, for example, the temperature experienced during development determines adult pigmentation and life histories (**Figure 1A**) and anticipates seasonal changes in background foliage coverage, favoring season-specific anti-predatory and reproductive strategies (Brakefield et al., 2009; Monteiro, 2015; Beldade and Peralta, 2017). In such seasonal polyphenisms, inductive and selective environments reflect temporal heterogeneity (Brakefield et al., 2007). External factors can also reflect spatial heterogeneity, such as that associated with different levels of predation between ponds inhabited by *Rana temporaria* tadpoles (Van Buskirk, 2017), with the alternative environmentally induced phenotypes being produced on different locations. In other cases of plasticity, the alternative morphs co-occur. For instance, with the nutritional-dependent caste determination in social insects, the heterogeneity in adult “environment,” rather than in any external factor, exists in terms of task allocation inside the colony (Jeanson and Weidenmüller, 2014; Gordon, 2016).

Typically, one same inductive cue will simultaneously affect different traits (e.g. predator presence affects head morphology and body size in *Poecilia reticulata* guppies; Torres-Dowdall et al., 2012), which are often part of the same “plasticity syndrome”. In these cases, different traits can be integrated into functional suites that respond to environmental influences in a concerted manner and that are typically also selected in concert (e.g. Mateus et al., 2014; Oostra et al., 2014; van Bergen and Beldade, 2019). Such extent of integration (or, conversely, independence) among plastic traits, some of which might be adaptive, whereas others might be maladaptive, has important implications for phenotypic variation and diversification as it influences responses to selection. A classical example of correlated plastic responses is the effect that temperature has on different phenotypes, including development time (e.g. diapause), body size, and other life-history traits in many arthropods. Although diapause is thought to be an adaptive plastic response, this may not be true for correlated traits whose developmental rates are affected by the availability of energy resources (Gotthard and Nylin, 1995). It is also common that one same trait can be simultaneously affected by different environmental cues (e.g. production of the winged morph in aphids affected by tactile stimulation, nutrition, and other factors; Braendle et al., 2006). Exploring how organisms integrate information from different external cues is a topic of much current interest, as studies of plasticity start to tackle what is the typical complexity of natural environments, where organisms are exposed to multiple and highly dynamic environmental factors (e.g. Saastamoinen et al., 2013; Fischer et al., 2017; Saxon et al., 2018).

### Reaction Norms

Reaction norms (**Figures 1B–D**), where variation in phenotype is displayed as a function of variation in environment, are a common and very useful way to graphically represent developmental plasticity (Schlichting and Pigliucci, 1998; DeWitt and Scheiner, 2004; Sultan, 2017). These representations reflect the extent and the effects of environmental cues on phenotype expression (**Figure 1B**). Horizontal (flat) lines represent traits that are invariant, or robust (Félix and Barkoulas, 2015), to environmental conditions. For example, vulval cell fate patterning in *Caenorhabditis elegans* has been shown to be unresponsive to changes in temperature, salinity, or nutrients, which affect other aspects of worm development (Félix, 2012). Diagonal lines, in contrast, reflect a gradual relationship between environment and phenotype, such as temperature-induced differences in insect body size (Nijhout, 2003b; Mirth and Shingleton, 2012); the steepest the line is, the more plastic is the trait. In other cases, such as the polyphenisms described earlier, responses to environmental variation can be discontinuous, with discrete phenotypes below and above some environmental thresholds (e.g. Pfennig, 1992; Kamakura, 2011; Casasa and Moczek, 2018). Well-described examples of non-linear reaction norms resulting in polyphenims include honey bees, where only larvae that are fed high quantities of royal jelly develop into queens (Kamakura, 2011), and the diet-dependent size of horns in dung beetle males, which is disproportionally large for large males (Moczek, 1998). Some of such discontinuous reaction norms may result from cases where organisms can only produce two alternative phenotypes or from cases where organisms have never been exposed to intermediate environmental conditions, which would otherwise reveal gradual reaction norms.

The degree and type of plastic responses can vary between species, as is the case with the density-dependent swarming responses in locusts and grasshoppers (Song et al., 2017) or with the thermal effects on pigmentation in different mycalesine butterflies (van Bergen et al., 2017). Plastic responses can also differ between populations of the same species (**Figures 1B, C**). For instance, the effects of oxygen on brain and gill size differ between populations of cichlid fish from different geographical locations (and oxygen regimes; Crispo and Chapman, 2010). Reaction norms typically describe how one environmental factor affects one specific trait for one particular genetic background, and they usually differ between traits, environmental cues, and genotypes. For instance, dung beetle horn size increases more with food quantity than does body size (Moczek, 2002; Casasa and Moczek, 2018). Different melanin-based traits respond in independent (sometimes even opposing) manners to temperature and photoperiod in butterflies (Stoehr and Wojan, 2016). The different responses that genotypes can have to environmental inputs correspond to non-parallel reaction norms and significant genotype-by-environment (GxE) interactions (Saltz et al., 2018; **Figures 1C, D**). The genes responsible for those inter-genotype differences in reaction norms can presumably fuel the evolution of plasticity and will be further discussed in *Genes for Variation in Plasticity*.

External environmental cues affect adult phenotype by altering developmental rates and/or trajectories. For this to happen, organisms must be able to somehow sense external conditions and provide information about those conditions to the developing tissues where changes in developmental cascades will result in changes in phenotype (**Figure 2A**). The cellular and molecular players involved in the sequence of steps that connect variation in external conditions to variation in developmental outcomes have been characterized in a number of cases, such as *dauer* formation in *C. elegans* nematodes (see Fielenbach and Antebi, 2008; Allen et al., 2015; **Figure 2B**). The perception of external conditions can involve specific neurons (e.g. temperature-sensitive neurons in *D. melanogaster*; Peng et al., 2007) and/or specific molecules (e.g. ascaroside pheromones that indicate high density in *C. elegans*; Ludewig and Schroeder, 2013). The information about the external conditions is then conveyed to the tissues developing internally, a process that typically involves one or various hormones whose synthesis, degradation, and/or activation depend on environmental conditions (Mirth et al., 2009; Mirth et al., 2014; Vellichirammal et al., 2017). These hormones will then affect gene expression and/or function in the target plastic tissues (e.g. Koyama et al., 2013; Monteiro et al., 2015). In some cases, direct effects of environmental factors on gene expression have been reported, for instance, with temperature presumably directly regulating the transcription of clock genes in zebrafish (Lahiri et al., 2005). Because developmental plasticity refers to the production of different phenotypes from the same genotype, it necessarily involves epigenetic mechanisms, i.e. that are beyond the nucleotide sequence in genomic DNA. Mechanisms such as methylation of DNA or acetylation of histones, for example, are capable of mediating changes in gene expression without changes in gene sequence and have been implicated in plastic development (e.g. caste determination in honeybees and ants; Kucharski et al., 2008; Lyko et al., 2010; Bonasio et al., 2012; Simola et al., 2013b, Simola et al., 2016). The cascade of events from environmental inputs to alternative phenotypes will involve “effector genes” (cf. **Figure 2**) whose expression and/or function depend on environmental conditions and underlie developmental changes. These effector genes will be further discussed in *Environmentally Responsive Genes*.

### Plasticity and Evolution

Theoretical and *in silico* studies have identified a series of conditions that can influence the evolution of plasticity (e.g. Tufto, 2000; Lande, 2009). These include the predictability of environmental fluctuations and the reliability of inductive environmental cues in predicting the future selective environment (Leimar et al., 2006; Reed et al., 2010), both of which should favor plasticity. The availability and effective assessment of such cues will determine the evolution of plasticity in relation to other possible responses to environmental variation, such as bet-hedging (Simons, 2011; Herman et al., 2014; Tufto, 2015). The evolution of plasticity can also be influenced by other factors, such as potential costs associated with maintaining the sensory and regulatory mechanisms needed for plastic responses (DeWitt, 1998; Tufto, 2000; Callahan et al., 2008; Snell-Rood, 2012; Lande, 2014; Murren et al., 2015). Trade-offs between plasticity and different fitness-related traits have been documented that can presumably constrain the evolution of plasticity. For example, in freshwater snails, the levels of predator-induced plasticity in shell shape have been shown to be negatively correlated with growth rate (DeWitt, 1998) and predator-induced phenotypes have been shown to have lower survival in the presence of a different predator (Hoverman and Relyea, 2009). When the ecological conditions favor plasticity, and because plasticity is itself a heritable trait (Via and Lande, 1985; Scheiner and Lyman, 1989, Scheiner and Lyman, 1991; Thompson, 1991; Carter et al., 2017), plasticity can and does evolve (**Box 1**). Several studies have documented transitions between plastic and robust development in both natural populations (e.g. Cahan et al., 2004; Aubret and Shine, 2009) and laboratory populations (e.g. Suzuki and Nijhout, 2006; Bento et al., 2010). The loci carrying allelic variation responsible for variation in plasticity that can fuel its evolution will be discussed in *Genes for Variation in Plasticity*.

It is believed that, in most cases, the ancestral condition in trait development is some level of environmental sensitivity, with selection then favoring the ability to buffer environmental effects (Newman and Müller, 2000; Nijhout, 2003a; Nijhout et al., 2017). Selection can act on the regulation of environmentally sensitive phenotypes and adjust the properties and/or magnitude of the plastic responses by a process called genetic accommodation (West-Eberhard, 2003; West-Eberhard, 2005; Crispo, 2007). Well-documented examples include the evolution of thermal plasticity in larval pigmentation in experimental populations of *Manduca sexta* (Suzuki and Nijhout, 2006) and the evolution of developmental rate in response to aridification in spadefoot toads (Gomez-Mestre and Buchholz, 2006; Kulkarni et al., 2017). When genetic accommodation leads to the (genetic) fixation of what was previously an environmentally induced phenotype, we talk about genetic assimilation (Schlichting and Wund, 2014; Ehrenreich and Pfennig, 2016; Schneider and Meyer, 2017). The occurrence and underlying genetic mechanism of genetic assimilation have what is arguably its classical example in the fixation of a *bithorax* phenotype originally induced by the exposure of *D. melanogaster* to ether (Waddington, 1953; Gibson and Hogness, 1996). More recent studies of genetic assimilation have illustrated how transitions from environmental to genetic control of adaptive traits may happen at a (relatively) fast pace (e.g. loss of plasticity in head size in *Notechis scutatus* snakes within a few thousand years; Aubret and Shine, 2009) and how it can lead to complex interdependence between environmentally and genetically induced phenotypes (e.g. with caste determination in *Pogonomyrmex* harvester ants; Cahan et al., 2004).

Whereas, in the previous section, we discussed how plasticity is a trait under selection and can evolve, here we will focus on how plasticity might impact adaptive evolution (**Box 1**). Evolved plasticity is thought to be able to help populations face challenges posed by changing environments (e.g. de Jong, 2005; Xue and Leibler, 2018; Fox et al., 2019), allow them to cope with environmental heterogeneity (e.g. West-Eberhard, 2003; Lande, 2009; Levis et al., 2018), and aid the colonization of novel environments (e.g. Lande, 2015; Snell-Rood et al., 2018). More than plasticity potentially enabling persistence under novel conditions and thereby “buying time” for adaptation to occur (Corl et al., 2018; Pennisi, 2018), it has been suggested that plasticity may actually promote adaptive evolution and diversification (e.g. see Wund, 2012; Smith and Ritchie, 2013; Gilbert et al., 2015). This idea has been proposed with a number of variations, sometimes subtle, that emphasize different mechanisms and/or outcomes. Modeling work has sustained that plasticity might be able to foster an increase in the levels of genetic variation in a population (e.g. Draghi and Whitlock, 2012), thereby impacting adaptive evolution. A particular type of genetic variation often associated with plasticity is called cryptic genetic variation, i.e. genetic variation that is normally not expressed but can be uncovered by environmental or genetic perturbation (e.g. Gibson and Dworkin, 2004; McGuigan et al., 2011; Paaby and Rockman, 2014; Schneider and Meyer, 2017). Plasticity has been argued to favor both the accumulation of cryptic genetic variation (e.g. in genes not expressed under certain environmental conditions) and its release (e.g. upon environmental conditions outside the range typically experienced by the population). Such genetic variation can then fuel evolutionary change (Gibson and Dworkin, 2004; Ledón-Rettig et al., 2010; Paaby and Rockman, 2014; Schneider and Meyer, 2017). Furthermore, the evolutionary potential of plasticity has been illustrated by the contribution of plasticity to reproductive isolation, which includes cases where plastic traits affect mating (e.g. pigmentation patterns in butterflies; Westerman et al., 2014) or timing of organismal life-events (e.g. phenological shifts in grasshoppers; Buckley et al., 2015).

The range of phenotypic variation generated by plasticity has been proposed to be able to initiate and accelerate the pace of adaptive evolution and to promote morphological as well as phylogenetic diversification (West-Eberhard, 2003, West-Eberhard, 2005; Pfennig et al., 2010; Wund, 2012). This relies on the suggestion that plasticity could be an immediate source of initial inter-individual differences in phenotypes (and in fitness) that could then become genetically fixed by genetic assimilation (West-Eberhard, 2003; Wund et al., 2008; Susoy et al., 2015; Levis and Pfennig, 2016; Gibert, 2017; Levis and Pfennig, 2019), leading to increased phenotypic diversification among populations. This same idea is at the basis of what have been called the “plasticity-first” model (Levis and Pfennig, 2016; Levis and Pfennig, 2018) and the “flexible stem” hypothesis, with the latter specifically suggesting that a “plastic ancestor” more readily originates phylogenetic divergence and adaptive radiations (West-Eberhard, 2003; Standen et al., 2014; Schneider and Meyer, 2017). This distinction between the two models is not always made; the terms have sometimes been used inter-changeably but are also often discussed separately (Wund et al., 2008; Muschick et al., 2011; Levis and Pfennig, 2016; Gibert, 2017). For instance, the invasion of new niches by amphibian species is proposed to represent a natural example of the “plasticity first” model, with nutrition-induced plasticity fostering the origin of carnivore morphs in some species (Levis et al., 2018). In contrast, evolutionary diversifications in threespine stickleback fish (with head and mouth shape variation across ecotypes; Wund et al., 2008) and tetrapods (with the origin of “terrestrialization” traits; Standen et al., 2014) have been discussed as examples supporting the “flexible stem” hypothesis. The study of plasticity and its (potential) impact on adaptive evolution has generated substantial interest but also much discussion (e.g. Pigliucci, 2007; Laland et al., 2014; Forsman, 2015; Nicoglou, 2015; Turcotte and Levine, 2016; Charlesworth et al., 2017; Futuyma, 2017). This includes controversy about definitions for plasticity as well as some level of lack of clarity about distinction and integration of different models proposed to account for the role of plasticity in adaptation and diversification.

## Genomics of Plasticity

Development can either respond to or resist environmental perturbation and the balance between such plasticity and/or robustness is crucial for organismal fitness. We know about different molecular mechanisms involved in buffering effects of environmental variation (i.e. conferring robustness to development; see Nijhout et al., 2017), including redundancy in gene enhancers (Frankel et al., 2010) and error-correcting systems (e.g. heat shock proteins; Queitsch et al., 2002; Sangster et al., 2004). Plasticity, on the other hand, has been proposed to be eased by modularity in molecular networks (Snell-Rood et al., 2010) and/or by expansion of certain gene families/classes some textbook models of plasticity, such as water fleas, aphids, and ants and other social insects (e.g. International Aphid Genomics Consortium, 2010; Colbourne et al., 2011; Wurm et al., 2011; Simola et al., 2013a).

**Figure 3:**
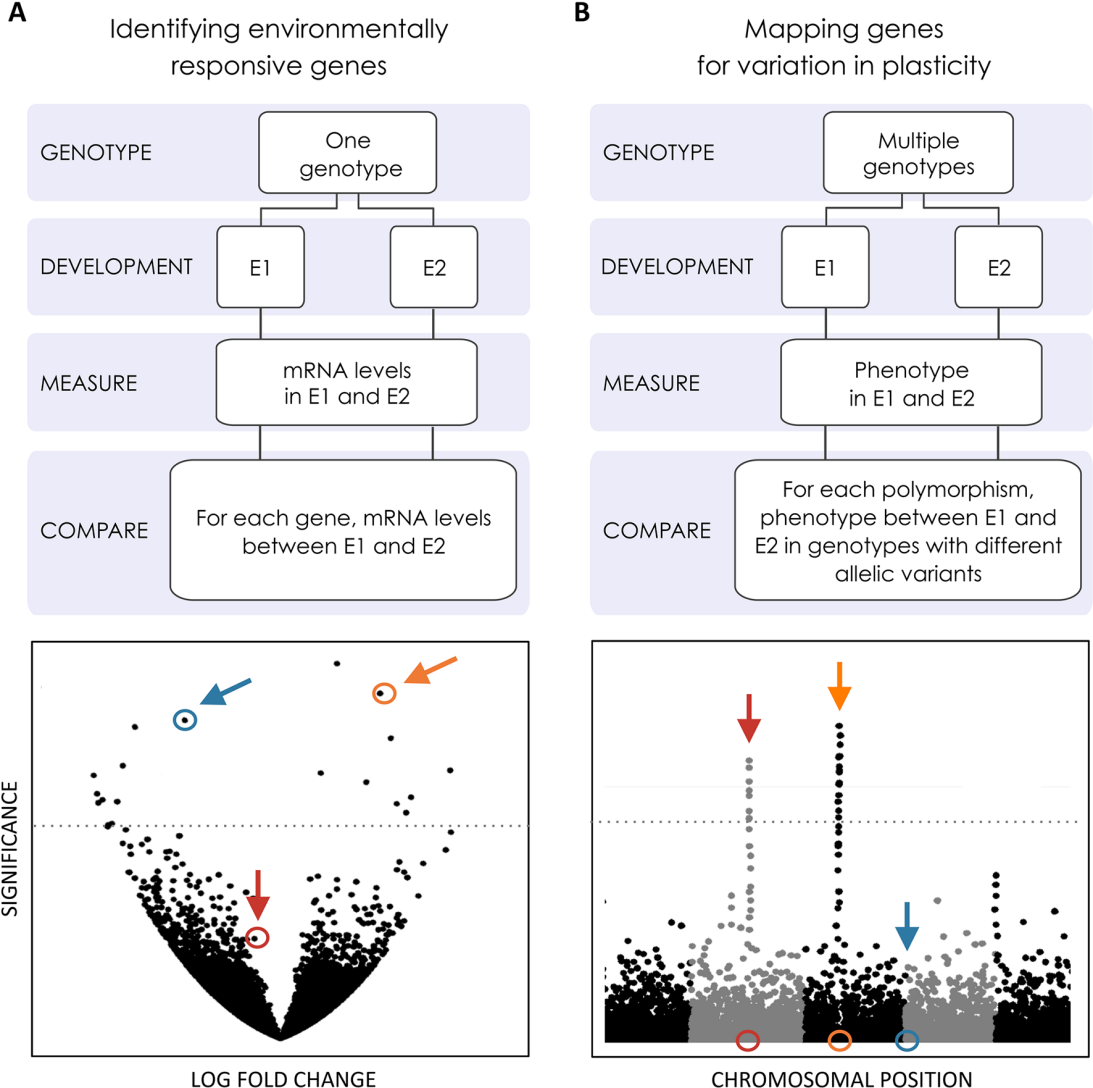
Genome-wide searches for the genetic basis of plasticity. Efforts to explore the genomics of plasticity include transcriptomics and mapping studies [e.g. genome-wide association studies (GWAS)] to identify genes whose expression changes across environments and loci contributing to variation in plasticity, respectively. These genes might or might not be the same, as illustrated by the colored loci in **(A** and **B)**. **(A)** Volcano plot where the significance in gene expression differences (*Y*-axis) is displayed as a function of fold change expression across environments (*X*-axis). Two genes showing significant expression differences (above significance threshold represented by the horizontal line) are highlighted in blue and orange. One where expression is not statistically significantly difference is highlighted in red. **(B)** Manhattan plot where statistical significance in the association with inter-genotype differences in reaction norms (*Y*-axis) is displayed for each polymorphic site along the chromosomes (*X*-axis). The location of two polymorphisms showing statistically significant association with plasticity variation is highlighted in red and orange, respectively. One below the statistical significance threshold is highlighted in blue. Genes whose expression is plastic across environments may (gene in orange) or may not (gene in blue) harbor allelic variants contributing to variation in plasticity. Conversely, genes associated with variation in plasticity may (gene in orange) or may not (gene in red) differ in expression between environments.

Developmental plasticity refers to the effect of environmental conditions on developmental outcomes; as such, it pertains to the environmental component of the total phenotypic variation that exists for a trait (**Figure 2C**). Yet, there is obviously a genetic basis for plasticity and studies that have attempted to tackle it from different angles (e.g. Tétard-Jones et al., 2011; Projecto-Garcia et al., 2017; Wellband and Heath, 2017; **Figure 3**). In this section, we focus on recent studies using gene-candidate and genome-wide approaches to unravel the genomics of plasticity. We will distinguish between two categories of genes: genes whose expression and/or function change across environments to affect developmental outcome (i.e. the effectors genes in **Figure 2**) and genes harboring allelic variants responsible for inter-genotype differences in reaction norms (as illustrated in **Figure 1C**). Although this distinction separates genes whose expression/function is environmentally dependent at the organismal level and loci whose effects determine differences in plasticity at the population level, the actual genes in the two categories can overlap to a certain extent (**Figure 3**).

### Environmentally Responsive Genes

Context-dependent gene expression is at the heart of development, transforming a single cell into spatially organized different cell types that make up multicellular organisms (Gilbert, 2000). In the case of developmental plasticity, the environmental regulation of development requires gene expression and/or function to depend also on external context. Here, we will focus on effector genes whose expression changes in plastic tissues to alter developmental fate (**Figure 2**). Efforts to identify such genes have documented differences in expression between environments for whole developing organisms or specific developing organs and either targeting specific candidate genes/pathways or doing transcriptome-wide scans (**Figure 3A**).

Candidate gene approaches to probe the genetic basis of environmentally regulated development have ranged from studies of gene expression levels (e.g. using quantitative polymerase chain reaction; e.g. Dalton et al., 2015) to studies of spatial expression patterns on specific organs (e.g. using *in situ* hybridization methods; Gibert et al., 2016). They have also included both *in vivo* (e.g. kairomone-driven regulation of patterning genes during production of the anti-predator defense morph in *Daphnia* water fleas; Miyakawa et al., 2010) and *in vitro* (e.g. temperature-dependent expression of sex-determining genes in turtle gonad cultures; Shoemaker-Daly et al., 2010) approaches as well as single-species (e.g. allatotropin expression under starvation in *M. sexta*; Lee and Horodyski, 2006) and multi-species (e.g. wing-patterning genes in polyphenic ants; Abouheif and Wray, 2002) analyses. Detailed studies of specific and well-known developmental pathways in emblematic plasticity models, such as pigmentation in *Drosophila* (e.g. De Castro et al., 2018), body and organ size in different insects (e.g. Lee and Horodyski, 2006; Mendes and Mirth, 2016), and wing development in ants (e.g. Abouheif and Wray, 2002), have been very insightful. The systematic investigation of melanogenesis enzymes underlying pigmentation development in *D. melanogaster* revealed how the environmental regulation of gene expression can happen *via* modulation of enhancer activity (Gibert et al., 2016; Gibert et al., 2017b; De Castro et al., 2018) or *via* effects on chromatin regulation (Gibert et al., 2007). The investigation of the hormonal mechanisms that underlie the nutritional regulation of body and organ size in different insects, on the other hand, demonstrated tissue and stage specificity of the effects of nutrition on the expression and sensitivity of several players from the insulin and target of rapamycin pathways (Mirth and Shingleton, 2014; Koyama et al., 2016; Mendes and Mirth, 2016). Finally, the analysis of the expression of wing development genes in workers versus queens in different ant species revealed dissociation (and modularity) of wing-patterning genes, which presumably influenced the evolutionary lability of this polyphenism (Abouheif and Wray, 2002; Béhague et al., 2018).

Genome-wide scans of different types have allowed for a more unbiased search of (putative) effector genes, including for traits for which the underlying developmental genetic basis is not well understood. Transcriptomic differences due to differences in developmental environments have been characterized for various species and in relation to various environmental cues, for instance, nutrition in beetles (Kijimoto et al., 2014) and ant castes (Evans and Wheeler, 2000), temperature in fish (Wellband and Heath, 2017), and multiple environmental cues in flies (Zhou et al., 2012). External environmental factors can affect the expression of a large number of genes, with 15% of the *D. melanogaster* genome being differentially expressed (Zhou et al., 2012) and 10% of the expressed genes being differentially spliced (Jakšić and Schlötterer, 2016) between temperatures. Aside from providing valuable quantitative insights into the distribution of environmental effects on gene expression levels (e.g. Zhou et al., 2012) and allowing researchers to draw transcriptomic reaction norms (Aubin-Horth and Renn, 2009; Gao et al., 2015; Oomen and Hutchings, 2017), transcriptomic scans have been very valuable at identifying candidate effector genes or pathways (see **Figure 3**) for further detailed analysis, which can be more or less obvious from the onset. Examples include specific gene classes/families differently expressed between castes in social insects (Evans and Wheeler, 2000), the down-regulation of growth and metabolism genes influencing the duration of larval stage in *Strongylocentrotus droebachiensis* sea urchins under food scarcity (Carrier et al., 2015), the differential expression of endocrine and pigmentation-related genes that underlie the seasonal pigmentation morphs in *Junonia coenia* butterflies (Daniels et al., 2014), and the caste-specific expression of chemoreception genes in termites (Mitaka et al., 2016). Whereas most studies have used transcriptomic approaches (e.g. Levine et al., 2011; Zhou et al., 2012; Chen et al., 2015) to investigate environmental effects on the levels and regulation of mRNAs and different types of non-coding RNAs (e.g. Garrett and Rosenthal, 2012; Jakšić and Schlötterer, 2016; Healy and Schulte, 2019), others have focused on environmentally induced changes in levels of protein (using proteomic approaches; e.g. Silvestre et al., 2012) and metabolites (using metabolomic approaches; see Bundy et al., 2009). Although such whole-genome scans can provide valuable insights onto the magnitude and nature of environmental effects on gene expression, it is crucial to remember that the identified differences in gene-product quantity do not necessarily translate into differences in organismal phenotypes (see Evans, 2015).

### Genes for Variation in Plasticity

The environmental sensitivity of development is a property of a genotype (e.g. Gockel et al., 2002; Nussey et al., 2005; Lardies, 2008; Saltz et al., 2018). For any specific trait and cue, genotypes can differ in various properties of the corresponding reaction norms (**Figure 1D**). This includes variation in intercept (e.g. thermal reaction norms for life-history traits in *Ischnura elegans* damselflies; Bouton et al., 2011), shape (e.g. thermal reaction norms for pigmentation in *Drosophila mediopunctata* flies; Rocha et al., 2009), and slope (e.g. thermal reaction norms for growth rate in *Orchesella cincta* springtails; Driessen et al., 2007). Genotypes can also differ in the environmental cue that triggers change or in the environmental threshold for the induction of phenotypic change (e.g. for hormesis in *C. elegans*; Sikkink et al., 2014). The genes responsible for variation in reaction norms provide the raw material for selection to drive the evolution of plasticity (see DeWitt and Scheiner, 2004). There is evidence for both the polygenic nature of artificially selected changes in shape and height of reaction norms (e.g. Wijngaarden and Brakefield, 2000) and the single allelic variants that cause loss (e.g. Bento et al., 2010; Ragsdale et al., 2013; Ragsdale and Ivers, 2016) or gain (e.g. Suzuki and Nijhout, 2006) of environmental sensitivity.

**Figure 4:**
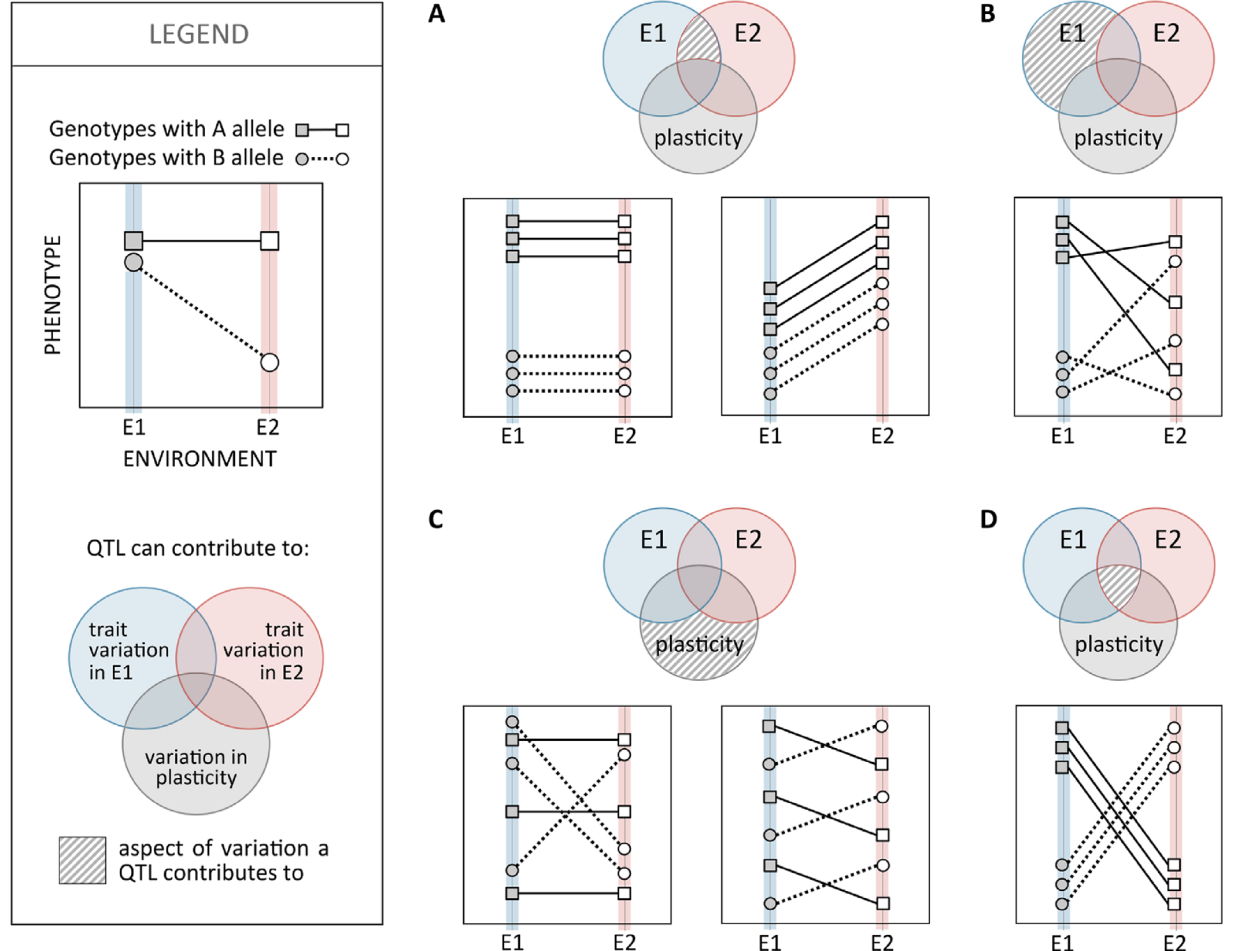
Relationship between quantitative trait loci (QTL) effects on trait mean and trait plasticity. Schematic representation of the ways in which a bi-allelic polymorphic site, e.g. a single nucleotide polymorphism (SNP) with alternative alleles (A and B; squares and circles, respectively), can contribute to phenotypic variation within fixed environmental conditions (E1 or E2; filled and empty symbols, respectively) and/or to plasticity in relation to those environmental values (reaction norms; solid and dashed lines). The contribution of each polymorphic site to phenotypic variation within environments and/or to variation in plasticity is illustrated in the Venn diagram. The area of the Venn diagram with a striped pattern indicates the aspect(s) of phenotypic variance a polymorphic site would be significantly associated with (i.e. is a QTL for). **(A)** Genotypes with the A allele have higher trait values than those with the B allele, in both environments E1 and E2, but reaction norms are of the same slope (corresponding to no plasticity in the left and to plasticity in the right). Such SNP would be associated with inter-genotype variation in environments E1 and E2 but not to inter-genotype variation in plasticity. **(B)** Genotypes with the A allele have higher trait values than those with the B allele in environment E1 but not in environment E2. Genotypes with A and B alleles have reaction norms that can be flat or steep in any direction. Such SNP could be associated with inter-genotype trait variation in environment E1 but not trait variation in environment E2 or variation in plasticity. **(C)** Genotypes with A and B alleles have same mean trait values in both environments E1 and E2 but different reaction norm properties: flat (genotypes with the A allele) versus steep (B allele) reaction norms on the left and positive (A allele) versus negative (B allele) reaction norms on the right. Such SNPs would be associated with variation in plasticity but not variation within E1 or E2. **(D)** An example of an SNP affecting both trait variation in E1 (A alleles corresponding to higher trait values than B allele) and E2 (A alleles corresponding to lower trait values than B allele) as well as variation in plasticity (negative slope reaction norms for A allele versus positive for B allele).

To identify loci contributing to inter-genotype variation in plasticity, researchers have compared reaction norms between allelic variants of specific candidate genes or used genome-wide mapping scans of different types. Studies of thermal plasticity in *D. melanogaster* development, for example, illustrate both approaches: differences in reaction norms for abdominal pigmentation for mutant versus wild-type alleles of different melanogenesis genes (Gibert et al., 2007; Gibert et al., 2017b) and mapping of loci with allelic variation associated with variation in the slope of reaction norms for body size (Lafuente et al., 2018). Although, in the past couple of decades, we have seen great progress in unraveling the genetic basis of phenotypic variation and adaptation, covering many different traits and species (e.g. Barrett and Hoekstra, 2011; Martin and Orgogozo, 2013; Savolainen et al., 2013; Pardo-Diaz et al., 2015), relatively little is known about the genetic basis of variation in plasticity (see Pigliucci, 2005). In fact, most quantitative trait loci (QTL) mapping studies have tracked phenotypic variation under one single environmental condition, precluding the assessment of environment-specific or gene-by-environment effects. Studies that did include phenotyping under different environments (e.g. survival after heat stress in zebrafish; Hosseini et al., 2018), as is necessary to characterize the genetic basis of variation in plasticity, revealed a prevalence for environment-specific QTL (Dilda and Mackay, 2002; Green et al., 2014), which are QTL that are expressed differently in different environments. Studies mapping variation in *D. melanogaster* at different temperatures showed large proportions of QTLs exhibiting QTL-by-environment interactions, for example, 70% (in Gurganus et al., 1998) and 33% to 50% (in Dilda and Mackay, 2002) for bristle number and 83% for reproductive performance (Fry et al., 1998). Environment-specific QTL also includes cases with alleles having antagonistic effects in different environments (e.g. up to 59% of QTLs for variation in life span at different temperatures and nutritional regimes in *D. melanogaster*; Vieira et al., 2000). Although QTL-by-environment interactions certainly reflect inter-genotype differences in reaction norms, they are not necessarily QTLs contributing to inter-genotype variation in plasticity (**Figure 4**). To unravel such loci, mapping has used the slope of reaction norms as the target quantitative trait. Examples include the identification of QTLs associated with the slope of reaction norms for thermal regulation of life-history traits in *C. elegans* (Gutteling et al., 2007) and of body size (Lafuente et al., 2018) and cold tolerance (Ørsted et al., 2018) in *D. melanogaster*. These studies identified QTLs corresponding to different types of genomic regions (e.g. regulatory and coding) and different putative biological functions (e.g. regulation of development, components of the nervous system, and environmental stress responsive genes), including regulatory genes (Ørsted et al., 2018), which are thought to play key roles on the genetic control of plasticity (Schlichting and Pigliucci, 1993).

More than identifying specific genes and describing their function, studies of the genetic basis of plasticity have shed light onto old debates about the existence and nature of plasticity genes (Via, 1993; DeWitt and Scheiner, 2004; Nicoglou, 2015). The proposed models had distinguished between scenarios where plasticity is a side effect of selection on the (plastic) trait versus where plasticity is itself a direct target of selection (Via and Lande, 1985; Scheiner and Lyman, 1991; Schlichting and Pigliucci, 1993;). Whereas positive genetic correlations between trait values and trait plasticity and environmentally dependent allelic effects are presumably consistent with the former (e.g. Fry et al., 1998), examples where selection on plasticity is independent of selection on trait mean (e.g. thermal plasticity in the timing of egg-laying in *Ficedula albicollis* collared flycatchers; Brommer et al., 2005, and in thorax size in *D. melanogaster*; Scheiner and Lyman, 1991) and/or where selection on trait mean does not constrain plastic responses (e.g. for cold tolerance in *B. anynana* butterflies; Franke et al., 2012) are consistent with the latter. In fact, several recent mapping studies have reported little overlap between genes contributing to variation in trait means with those contributing to variation in trait plasticity (e.g. Lafuente et al., 2018; Ørsted et al., 2018). This suggests a distinct genetic basis for trait plasticity versus the genetic basis for phenotypic variation in the trait itself (at any given environment) and points to the potential for independent evolution of the two.

### Integration and Challenges

Several studies in animals have combined the high-throughput gene expression analysis under different environments with forward genetic approaches to identify the genetic basis underlying environmentally dependent gene expression differences (Runcie et al., 2012). These studies, which involve assessing gene expression under different environmental conditions in different genotypes [for the so-called eQTL analysis or genome-wide association studies (GWAS) of gene expression; e.g. Li et al., 2006], allowed to link gene expression differences to genomic markers or allelic variants (see Grishkevich and Yanai, 2013). By associating variation in genotype to variation in environmentally regulated gene expression, researchers have not only characterized GxE interactions in gene expression and their underlying genetic basis but also identified genomic features distinguishing genes with GxE interactions from other types of genes. Work in *C. elegans*, for example, revealed that genes that exhibited GxE interactions tend to show distinct promoter architecture (e.g. long promoter with a high concentration of regulatory motifs) as well as mid-range expression level (Grishkevich et al., 2012), both of which are characteristic of tightly regulated genes (Promislow, 2005).

We have explicitly focused on genomic work studying genes involved in the regulation of plastic responses, i.e. those that respond to environmental inputs and affect trait expression, and genes potentially involved in the evolution of plastic responses, i.e. those that harbor allelic variants contributing to differences in reaction norms. There is, of course, some overlap in the genes involved in trait development, trait plasticity, and plasticity variation (e.g. orange gene in **Figure 3**). A compelling example comes from studies of abdominal pigmentation in *Drosophila*. The gene *tan*, encoding one of the melanogenesis enzymes (True, 2003), has been implicated in inter-individual variation in pigmentation within a given temperature (e.g. allelic variants of *tan* underlie differences in pigmentation; Dembeck et al., 2015), regulation of pigmentation plasticity (e.g. flies with reduced expression of *tan* have lighter pigmentation; Kalay et al., 2016), and variation in pigmentation plasticity (e.g. different *tan* alleles correspond to different reaction norms; Gibert et al., 2016). However, there does not need to be a complete overlap between the genes involved in plasticity regulation and those that contribute to plasticity variation/evolution (**Figure 3**). Indeed, a gene that is differentially expressed between developmental environments might not harbor allelic variants contributing to variation in reaction norms (e.g. blue in **Figure 3**). Similarly, a gene contributing to variation in reaction norms does not need to differ in expression across environments (e.g. red in **Figure 3**).

In the previous sections, we highlighted different genomic studies to identify the genes involved in the regulation and evolution of plasticity, including transcriptomics or genetic mapping approaches. Although extremely powerful at identifying candidate genes, these genome-wide scans are also very prone to false positives and not very informative about the actual role of those genes. As such, it is crucial to independently run functional analysis (Gibert et al., 2017a) to validate candidate genes, something not always easily accessible. Tools for the manipulation of gene function (e.g. transgenic knock-downs or knock-outs to reduce and abolish gene expression) as well as the manipulation of specific sites (e.g. *via* allelic replacements) were typically only easily accessible in established models. However, fast advances in analytical tools, for both genomic and functional studies, are now enabling us to move from model organisms to less established models (e.g. Marinković et al., 2012; Santure and Garant, 2018; see Russell et al., 2017). The progress in methods is also aiding researchers to move from laboratory strains to natural populations, often including more realistic scenarios in terms of the diversity in genetic backgrounds and demographic parameters and of the complexity of external environments. All these factors are bound to affect phenotype expression and/or evolution (Braendle and Félix, 2008; Chevin, 2013; Piggott et al., 2015; Bretman et al., 2016; Matsui and Ehrenreich, 2016; Fischer et al., 2017). How organisms perceive and integrate information from complex environments, with multiple factors that can vary more or less independently, including within the time it takes to complete development (Warkentin, 2011; Fischer et al., 2017; Rodrigues et al., 2017), is a fascinating topic in need of deeper characterization.

Studies investigating the genes underlying plastic responses are rapidly increasing. Previous research using a variety of systems illustrated that environmentally induced changes in gene expression are pervasive and can include a variety of molecular and functional genes classes (e.g. Zhou et al., 2012; Kijimoto et al., 2014), as reviewed here. Future work exploring changes in gene expression beyond a single species (or a single population), and beyond a single environmental cue, could potentially help to identify general patterns about the effector genes underlying developmental plasticity, whether they belong to particular functional classes or are shared between cues. Moreover, as much more is known about the molecular mechanisms and the genetics of environmentally induced phenotypes in plants (see Sultan, 2000; Sultan, 2017), these are exciting times to start integrating studies on plasticity from animals and plants, which could provide insights on the potential commonalities in the mechanisms controlling whether phenotypes would respond to (or buffer) environmental variation.

## Concluding Remarks

Developmental plasticity can result in a better match between adult phenotype and adult environment, thus helping organisms cope with environmental heterogeneity. An adaptive value for plasticity is compellingly illustrated by examples of seasonal polyphenisms, when the same genetic background produces phenotypes adjusted to the different conditions of alternating seasons as a response to environmental factors that anticipate those conditions (see Kivelä et al., 2013). Recent work has provided evidence that some phenotypes traditionally associated with environmental differences, such as those that vary seasonally, are in fact due to genetic differentiation under strong temporally variable selection (e.g. Bergland et al., 2014; Foucault et al., 2018). Examples such as these open up the possibility that other fast phenotypic changes assigned to plasticity could be due to rapid adaptation, an area that will certainly get increased future attention.

Plasticity can provide the means of rapidly adjusting to external change; for that reason, its study is getting increased attention in relation to the ability of organisms to deal with climate change (see Merilä and Hendry, 2014; Sgrò et al., 2016; Kelly, 2019). Plasticity may mitigate the negative effects that climate change can have on population persistence (Charmantier et al., 2008; Teplitsky et al., 2008; Kingsolver and Buckley, 2017; Burggren, 2018) by producing phenotypes better adjusted to the new climatic conditions (e.g. Przybylo et al., 2000; Réale et al., 2003; Gienapp et al., 2013) or by enabling plastic species (or populations) to track environmental changes and cope with a wider range of environments than non-plastic ones (e.g. Charmantier et al., 2008). Changes in breeding timing in mammals (e.g. *Tamiasciurus hudsonicus* red squirrels; Réale et al., 2003) and birds (e.g. *Parus major* great tits; Charmantier et al., 2008) represent examples in which plasticity has presumably contributed to phenotypic trends associated with contemporary climate change. However, plasticity may also make populations more vulnerable to climate change (e.g. Mills et al., 2013; Zimova et al., 2016), for instance, if the previously established association between inductive and selective environments is disrupted (Visser et al., 2010; Oostra et al., 2018) or in cases of species with temperature-dependent sex determination, when climate change can alter the hatchling sex ratio and survivorship and therefore impact population demographics and/or persistence (Fuentes et al., 2011; Jensen et al., 2018). Examples such as these will be valuable for assessing the role of plasticity in coping with climate or other types of global change.

Much has been written about the contribution of plasticity to evolution in recent years (e.g. Forsman, 2015; Gilbert et al., 2015; Turcotte and Levine, 2016; Schneider and Meyer, 2017; Levis and Pfennig, 2018). Controversially (e.g. Laland et al., 2014; Charlesworth et al., 2017; Futuyma, 2017), some authors defend a need for an “extended evolutionary synthesis” (Jablonka et al., 2014; Laland et al., 2015) to explicitly incorporate plasticity, as well as other aspects of organismal development, inheritance and fitness, into evolutionary models (Jablonka et al., 2014; Pigliucci, 2007; Pigliucci, 2009; Gissis and Jablonka, 2011; Laland et al., 2014, Laland et al., 2015). There is undoubtedly a recent increase in interest on the adaptive value of plasticity, its role in adaptation, and its genetic basis. We have summarized recent insights onto two aspects of the genetic basis of plasticity in animals: genes whose expression (and function) depends on environmental conditions and lead to changes in development and genes that harbor allelic variants associated with differences in plasticity between genotypes and provide the raw material for natural selection to drive the evolution of plasticity. Given the availability of sophisticated tools, it is now becoming accessible to explore the regulation and evolution of plasticity in natural populations that deal with complex environments. This is a fascinating area of research for which we can surely expect more insights in years to come.

## Author Contributions

EL and PB conceived and wrote the manuscript.

## Funding

Financial support for this work was provided by the Portuguese science funding agency, Fundação para a Ciência e Tecnologia, FCT: PhD fellowship to E.L. (SFRH/BD/52171/2013), and research support for P.B. (PTDC/BIA-EVF/0017/2014, and PTDC/BEX-BID/5340/2014).

## Conflict of Interest Statement

The authors declare that the research was conducted in the absence of any commercial or financial relationships that could be construed as a potential conflict of interest.

## References

[B1] Abbey-Lee R. N. Dingemanse N. J. (2019). Adaptive individual variation in phenological responses to perceived predation levels. Nat. Commun. 10, 1601. 10.1038/s41467-019-09138-5 30962485 PMC6453887

[B2] Abouheif E. Wray G. A. (2002). Evolution of the gene network underlying wing polyphenism in ants. Science 297, 249–252. 10.1126/science.1071468 12114626

[B3] Allen E. Ren J. Zhang Y. Alcedo J. (2015). Sensory systems: their impact on *C. elegans* survival. Neuroscience 296, 15–25. 10.1016/j.neuroscience.2014.06.054 24997267 PMC4282626

[B4] Aubin-Horth N. Renn S. C. P. (2009). Genomic reaction norms: using integrative biology to understand molecular mechanisms of phenotypic plasticity. Mol. Ecol. 18, 3763–3780. 10.1111/j.1365-294X.2009.04313.x 19732339

[B5] Aubret F. Shine R. (2009). Genetic assimilation and the postcolonization erosion of phenotypic plasticity in island tiger snakes. Curr. Biol. 19, 1932–1936. 10.1016/j.cub.2009.09.061 19879141

[B6] Barrett R. D. H. Hoekstra H. E. (2011). Molecular spandrels: tests of adaptation at the genetic level. Nat. Rev. Genet. 12, 767–780. 10.1038/nrg3015 22005986

[B7] Béhague J. Fisher B. L. Péronnet R. Rajakumar R. Abouheif E. Molet M. (2018). Lack of interruption of the gene network underlying wing polyphenism in an early-branching ant genus. J. Exp. Zool. Part B Mol. Dev. Evol. 330, 109–117. 10.1002/jez.b.22794 29504672

[B8] Beldade P. Mateus A. R. A. Keller R. A. (2011). Evolution and molecular mechanisms of adaptive developmental plasticity. Mol. Ecol. 20, 1347–1363. 10.1111/j.1365-294X.2011.05016.x 21342300

[B9] Beldade P. Peralta C. M. (2017). Developmental and evolutionary mechanisms shaping butterfly eyespots. Curr. Opin. Insect Sci. 19, 22–29. 10.1016/j.cois.2016.10.006 28521939

[B10] Bento G. Ogawa A. Sommer R. J. (2010). Co-option of the hormone-signalling module dafachronic acid-DAF-12 in nematode evolution. Nature 466, 494–497. 10.1038/nature09164 20592728

[B11] Bergland A. O. Behrman E. L. O’Brien K. R. Schmidt P. S. Petrov D. A. (2014). Genomic evidence of rapid and stable adaptive oscillations over seasonal time scales in *Drosophila* . PLoS Genet. 10, e1004775. 10.1371/journal.pgen.1004775 25375361 PMC4222749

[B12] Bonasio R. Li Q. Lian J. Mutti N. S. Jin L. Zhao H. (2012). Genome-wide and caste-specific DNA methylomes of the ants *Camponotus floridanus* and *Harpegnathos saltator* . Curr. Biol. 22, 1755–1764. 10.1016/j.cub.2012.07.042 22885060 PMC3498763

[B13] Bossdorf O. Richards C. L. Pigliucci M. (2008). Epigenetics for ecologists. Ecol. Lett. 10.1111/j.1461-0248.2007.01130.x 18021243

[B14] Bouton N. Iserbyt A. Van Gossum H. (2011). Thermal plasticity in life-history traits in the polymorphic blue-tailed damselfly, *Ischnura elegans*: no differences between female morphs. J. Insect Sci. 11, 112. 10.1673/031.011.11201 22224863 PMC3281378

[B15] Braendle C. Davis G. K. Brisson J. A. Stern D. L. (2006). Wing dimorphism in aphids. Heredity (Edinb). 97, 192–199. 10.1038/sj.hdy.6800863 16823401

[B16] Braendle C. Félix M.-A. (2008). Plasticity and errors of a robust developmental system in different environments. Dev. Cell 15, 714–724. 10.1016/j.devcel.2008.09.011 19000836

[B17] Brakefield P. M. Beldade P. Zwaan B. J. (2009). The African butterfly *Bicyclus anynana:* a model for evolutionary genetics and evolutionary developmental biology. In Emerging Model Organisms: A Laboratory Manual. Eds. Behringer R. R. Johnson A. D. Krumlauf R. E. (New York: Cold Spring Harbor Laboratory Press), 291–329. 10.1101/pdb.emo122 20147150

[B18] Brakefield P. M. Pijpe J. Zwaan B. J. (2007). Developmental plasticity and acclimation both contribute to adaptive responses to alternating seasons of plenty and of stress in Bicyclus butterflies. J. Biosci. 32, 465–475. 10.1007/s12038-007-0046-8 17536166

[B19] Bretman A. Fricke C. Westmancoat J. D. Chapman T. (2016). Effect of competitive cues on reproductive morphology and behavioral plasticity in male fruitflies. Behav. Ecol. 27, 452–461. 10.1093/beheco/arv170 27004011 PMC4797378

[B20] Brommer J. E. Merilä J. Sheldon B. C. Gustafsson L. (2005). Natural selection and genetic variation for reproductive reaction norms in a wild bird population. Evolution (N. Y.) 59, 1362. 10.1554/04-561 16050111

[B21] Buckley L. B. Nufio C. R. Kirk E. M. Kingsolver J. G. (2015). Elevational differences in developmental plasticity determine phenological responses of grasshoppers to recent climate warming. Proc. Biol. Sci. 282, 20150441. 10.1098/rspb.2015.0441 26041342 PMC4590449

[B22] Bundy J. G. Davey M. P. Viant M. R. (2009). Environmental metabolomics: a critical review and future perspectives. Metabolomics 5, 3–21. 10.1007/s11306-008-0152-0

[B23] Burggren W. (2018). Developmental phenotypic plasticity helps bridge stochastic weather events associated with climate change. J. Exp. Biol. 221, jeb161984. 10.1242/jeb.161984 29748332

[B24] Cahan S. H. Julian G. E. Rissing S. W. Schwander T. Parker J. D. Keller L. (2004). Loss of phenotypic plasticity generates genotype-caste association in harvester ants. Curr. Biol. 14, 2277–2282. 10.1016/j.cub.2004.12.027 15620656

[B25] Callahan H. S. Maughan H. Steiner U. K. (2008). Phenotypic plasticity, costs of phenotypes, and costs of plasticity. Ann. N. Y. Acad. Sci. 1133, 44–66. 10.1196/annals.1438.008 18559815

[B26] Carrier T. J. King B. L. Coffman J. A. (2015). Gene expression changes associated with the developmental plasticity of sea urchin larvae in response to food availability. Biol. Bull. 228, 171–180. 10.1086/BBLv228n3p171 26124444 PMC4706744

[B27] Carroll S. B. (2000). Endless forms: the evolution of gene regulation and morphological diversity. Cell 101, 577–580. 10.1016/S0092-8674(00)80868-5 10892643

[B28] Carter M. J. Lind M. I. Dennis S. R. Hentley W. Beckerman A. P. (2017). Evolution of a predator-induced, nonlinear reaction norm. Proc. Biol. Sci. 284 (1861), 20170859. 10.1098/rspb.2017.0859 28835554 PMC5577476

[B29] Casasa S. Moczek A. P. (2018). The role of ancestral phenotypic plasticity in evolutionary diversification: population density effects in horned beetles. Anim. Behav. 137, 53–61. 10.1016/j.anbehav.2018.01.004

[B30] Chakir M. Chafik A. Gibert P. David J. R. J. R. (2002). Phenotypic plasticity of adult size and pigmentation in *Drosophila*: thermosensitive periods during development in two sibling species. J. Therm. Biol. 27, 61–70. 10.1016/S0306-4565(01)00016-X

[B31] Charlesworth D. Barton N. H. Charlesworth B. (2017). The sources of adaptive variation. Proc. Biol. Sci. 284 (1855). 10.1098/rspb.2016.2864 PMC5454256 28566483

[B32] Charmantier A. McCleery R. H. Cole L. R. Perrins C. Kruuk L. E. B. Sheldon B. C. (2008). Adaptive phenotypic plasticity in response to climate change in a wild bird population. Science. 320, 800–803. 10.1126/science.1157174 18467590

[B33] Chen J. Nolte V. Schlötterer C. (2015). Temperature-related reaction norms of gene expression: regulatory architecture and functional implications. Mol. Biol. Evol. 32, 2393–2402. 10.1093/molbev/msv120 25976350 PMC4540970

[B34] Chevin L.-M. (2013). Genetic constraints on adaptation to a changing environment. Evolution. 67, 708–721. 10.1111/j.1558-5646.2012.01809.x 23461322

[B35] Chevin L.-M. Lande R. (2009). When do adaptive plasticity and genetic evolution prevent extinction of a density-regulated population? Evolution. 64, 1143–1150. 10.1111/j.1558-5646.2009.00875.x 19863583

[B36] Colbourne J. K. Pfrender M. E. Gilbert D. Thomas W. K. Tucker A. Oakley T. H. (2011). The ecoresponsive genome of *Daphnia pulex* . Science 331, 555–561. 10.1126/science.1197761 21292972 PMC3529199

[B37] Corl A. Bi K. Luke C. Challa A. S. Stern A. J. Sinervo B. (2018). The genetic basis of adaptation following plastic changes in coloration in a novel environment. Curr. Biol. 28, 2970-2977.e7. 10.1016/j.cub.2018.06.075 30197088

[B38] Crispo E. (2007). The Baldwin effect and genetic assimilation: revisiting two mechanisms of evolutionary change mediated by phenotypic plasticity. Evolution (N. Y.) 61, 2469–2479. 10.1111/j.1558-5646.2007.00203.x 17714500

[B39] Crispo E. Chapman L. J. (2010). Geographic variation in phenotypic plasticity in response to dissolved oxygen in an African cichlid fish. J. Evol. Biol. 23, 2091–2103. 10.1111/j.1420-9101.2010.02069.x 20722894

[B40] Dalton B. E. Lu J. Leips J. Cronin T. W. Carleton K. L. (2015). Variable light environments induce plastic spectral tuning by regional opsin coexpression in the African cichlid fish, *Metriaclima zebra* . Mol. Ecol. 24, 4193–4204. 10.1111/mec.13312 26175094 PMC4532641

[B41] Daniels E. V. Murad R. Mortazavi A. Reed R. D. (2014). Extensive transcriptional response associated with seasonal plasticity of butterfly wing patterns. Mol. Ecol. 23, 6123–6134. 10.1111/mec.12988 25369871 PMC4545284

[B42] Day T. McLeod D. V. (2018). The role of phenotypic plasticity in moderating evolutionary conflict. Am. Nat. 192, 230–240. 10.1086/698170 30016156

[B43] De Castro S. Peronnet F. Gilles J.-F. Mouchel-Vielh E. Gibert J.-M. (2018). bric à brac (bab), a central player in the gene regulatory network that mediates thermal plasticity of pigmentation in *Drosophila melanogaster* . PLOS Genet. 14, e1007573. 10.1371/journal.pgen.1007573 30067846 PMC6089454

[B44] de Jong G. (2005). Evolution of phenotypic plasticity: patterns of plasticity and the emergence of ecotypes. N. Phytol. 166, 101–118. 10.1111/j.1469-8137.2005.01322.x 15760355

[B45] Deans C. Maggert K. A. Tefankjian A. Wakimoto B. T. (2015). What do you mean, “epigenetic”? Genetics 199, 887–896. 10.1534/genetics.114.173492 25855649 PMC4391566

[B46] Dembeck L. M. Huang W. Carbone M. A. Mackay T. F. C. (2015). Genetic basis of natural variation in body pigmentation in *Drosophila melanogaster* . Fly (Austin). 9, 75–81. 10.1080/19336934.2015.1102807 26554300 PMC4826111

[B47] DeWitt T. J. (1998). Costs and limits of phenotypic plasticity: tests with predator-induced morphology and life history in a freshwater snail. J. Evol. Biol. 11, 465–480. 10.1046/j.1420-9101.1998.11040465.x

[B48] DeWitt T. J. Scheiner S. M. (2004). Phenotypic plasticity: functional and conceptual approaches. Oxford: Oxford University Press.

[B49] Dilda C. L. Mackay T. F. C. (2002). The genetic architecture of *Drosophila* sensory bristle number. Genetics 162, 1655–1674. 12524340 10.1093/genetics/162.4.1655 PMC1462363

[B50] Draghi J. A. Whitlock M. C. (2012). Phenotypic plasticity facilitates mutational variance, genetic variance, and evolvability along the major axis of environmental variation. Evolution (N. Y.) 66, 2891–2902. 10.1111/j.1558-5646.2012.01649.x 22946810

[B51] Driessen G. Ellers J. Van Straalen N. M. (2007). Variation, selection and heritability of thermal reaction norms for juvenile growth in *Orchesella cincta* (Collembola: Entomobryidae). Eur. J. Entomol. 104, 39–46. 10.14411/eje.2007.006

[B52] Edelaar P. Jovani R. Gomez-Mestre I. (2017). Should I change or should I go? Phenotypic plasticity and matching habitat choice in the adaptation to environmental heterogeneity. Am. Nat. 190, 506–520. 10.1086/693345 28937819

[B53] Ehrenreich I. M. Pfennig D. W. (2016). Genetic assimilation: a review of its potential proximate causes and evolutionary consequences. Ann. Bot. 117, 769–779. 10.1093/aob/mcv130 26359425 PMC4845796

[B54] Ernst U. R. Van Hiel M. B. Depuydt G. Boerjan B. De Loof A. Schoofs L. (2015). Epigenetics and locust life phase transitions. J. Exp. Biol. 218, 88–99. 10.1242/jeb.107078 25568455

[B55] Evans J. D. Wheeler D. E. (2000). Gene expression and the evolution of insect polyphenisms. BioEssays 23, 62–68. 10.1002/1521-1878(200101)23:1<62::AID-BIES1008>3.0.CO;2-7 11135310

[B56] Evans T. G. (2015). Considerations for the use of transcriptomics in identifying the “genes that matter” for environmental adaptation. J. Exp. Biol. 218, 1925–1935. 10.1242/jeb.114306 26085669

[B57] Félix M.-A. (2012). *Caenorhabditis elegans* vulval cell fate patterning. Phys. Biol. 9, 045001. 10.1088/1478-3975/9/4/045001 22871570

[B58] Félix M.-A. Barkoulas M. (2015). Pervasive robustness in biological systems. Nat. Rev. Genet. 16, 483–496. 10.1038/nrg3949 26184598

[B59] Fielenbach N. Antebi A. (2008). *C. elegans* dauer formation and the molecular basis of plasticity. Genes Dev. 22, 2149–2165. 10.1101/gad.1701508 18708575 PMC2735354

[B60] Fischer S. Bohn L. Oberhummer E. Nyman C. Taborsky B. (2017). Divergence of developmental trajectories is triggered interactively by early social and ecological experience in a cooperative breeder. Proc. Natl. Acad. Sci. 114, E9300–E9307. 10.1073/pnas.1705934114 29078289 PMC5676887

[B61] Forsman A. (2015). Rethinking phenotypic plasticity and its consequences for individuals, populations and species. Heredity (Edinb). 115, 276–284. 10.1038/hdy.2014.92 25293873 PMC4815454

[B62] Foucault Q. Wieser A. Waldvogel A.-M. Feldmeyer B. Pfenninger M. (2018). Rapid adaptation to high temperatures in *Chironomus riparius* . Ecol. Evol. 8 (24), 12780–12789. 10.1002/ece3.4706 30619582 PMC6308882

[B63] Fox R. J. Donelson J. M. Schunter C. Ravasi T. Gaitán-Espitia J. D. (2019). Beyond buying time: the role of plasticity in phenotypic adaptation to rapid environmental change. Philos. Trans. R. Soc. Lond. B. Biol. Sci. 374, 20180174. 10.1098/rstb.2018.0174 30966962 PMC6365870

[B64] Fraimout A. Jacquemart P. Villarroel B. Aponte D. J. Decamps T. Herrel A. (2018). Phenotypic plasticity of *Drosophila suzukii* wing to developmental temperature: implications for flight. J. Exp. Biol. 221, jeb166868. 10.1242/jeb.166868 29987053

[B65] Franke K. Dierks A. Fischer K. (2012). Directional selection on cold tolerance does not constrain plastic capacity in a butterfly. BMC Evol. Biol. 12, 235. 10.1186/1471-2148-12-235 23217138 PMC3538507

[B66] Frankel N. Davis G. K. Vargas D. Wang S. Payre F. Stern D. L. (2010). Phenotypic robustness conferred by apparently redundant transcriptional enhancers. Nature 466, 490–493. 10.1038/nature09158 20512118 PMC2909378

[B67] Fry J. D. Nuzhdin S. V. Pasyukova E. G. Mackay T. F. (1998). QTL mapping of genotype-environment interaction for fitness in *Drosophila melanogaster* . Genet. Res. 71, 133–141. 10.1017/S0016672398003176 9717436

[B68] Fuentes M. M. P. B. Limpus C. J. Hamann M. (2011). Vulnerability of sea turtle nesting grounds to climate change. Glob. Chang. Biol. 17, 140–153. 10.1111/j.1365-2486.2010.02192.x

[B69] Futuyma D. J. (2017). Evolutionary biology today and the call for an extended synthesis. Interface Focus 7, 20160145. 10.1098/rsfs.2016.0145 28839919 PMC5566807

[B70] Fuxjäger L. Wanzenböck S. Ringler E. Wegner K. M. Ahnelt H. Shama L. N. S. (2019). Within-generation and transgenerational plasticity of mate choice in oceanic stickleback under climate change. Philos. Trans. R. Soc. B. Biol. Sci. 374, 20180183. 10.1098/rstb.2018.0183 PMC6365864 30966960

[B71] Gao L. Geng Y. Yang H. Hu Y. Yang J. (2015). Gene expression reaction norms unravel the molecular and cellular processes underpinning the plastic phenotypes of *Alternanthera philoxeroides* in contrasting hydrological conditions. Front. Plant Sci. 6, 991. 10.3389/fpls.2015.00991 26617628 PMC4641913

[B72] Gapp K. Jawaid A. Sarkies P. Bohacek J. Pelczar P. Prados J. (2014). Implication of sperm RNAs in transgenerational inheritance of the effects of early trauma in mice. Nat. Neurosci. 17, 667–669. 10.1038/nn.3695 24728267 PMC4333222

[B73] Garrett S. C. Rosenthal J. J. C. (2012). A role for A-to-I RNA editing in temperature adaptation. Physiology (Bethesda). 27, 362–369. 10.1152/physiol.00029.2012 23223630 PMC4208822

[B74] Ghalambor C. K. McKay J. K. Carroll S. P. Reznick D. N. (2007). Adaptive versus non-adaptive phenotypic plasticity and the potential for contemporary adaptation in new environments. Funct. Ecol. 21, 394–407. 10.1111/j.1365-2435.2007.01283.x

[B75] Ghosh S. M. Testa N. D. Shingleton A. W. (2013). Temperature-size rule is mediated by thermal plasticity of critical size in *Drosophila melanogaster* . Proc. Biol. Sci. 280, 20130174. 10.1098/rspb.2013.0174 23595269 PMC3652456

[B76] Gibert J.-M. (2017). The flexible stem hypothesis: evidence from genetic data. Dev. Genes Evol. 227, 297–307. 10.1007/s00427-017-0589-0 28780641

[B77] Gibert J.-M. Blanco J. Dolezal M. Nolte V. Peronnet F. Schlötterer C. (2017a). Strong epistatic and additive effects of linked candidate SNPs for *Drosophila* pigmentation have implications for analysis of genome-wide association studies results. Genome Biol. 18, 126. 10.1186/s13059-017-1262-7 28673357 PMC5496195

[B78] Gibert J.-M. Peronnet F. Schlötterer C. (2007). Phenotypic plasticity in *Drosophila* pigmentation caused by temperature sensitivity of a chromatin regulator network. PLoS Genet. 3, 0266–0280. 10.1371/journal.pgen.0030030 PMC1797818 17305433

[B79] Gibert J.-M. Mouchel-Vielh E. De Castro S. Peronnet F. (2016). Phenotypic plasticity through transcriptional regulation of the evolutionary hotspot gene tan in *Drosophila melanogaster* . PLoS Genet. 12, e1006218. 10.1371/journal.pgen.1006218 27508387 PMC4980059

[B80] Gibert J.-M. Mouchel-Vielh E. Peronnet F. (2017b). Modulation of yellow expression contributes to thermal plasticity of female abdominal pigmentation in *Drosophila melanogaster* . Sci. Rep. 7, 43370. 10.1038/srep43370 28230190 PMC5322495

[B81] Gibson G. Dworkin I. (2004). Uncovering cryptic genetic variation. Nat. Rev. Genet. 5, 681–690. 10.1038/nrg1426 15372091

[B82] Gibson G. Hogness D. S. (1996). Effect of polymorphism in the *Drosophila* regulatory gene Ultrabithorax on homeotic stability. Science 271, 200–203. 10.1126/science.271.5246.200 8539619

[B83] Gienapp P. Lof M. Reed T. E. McNamara J. Verhulst S. Visser M. E. (2013). Predicting demographically sustainable rates of adaptation: can great tit breeding time keep pace with climate change? Philos. Trans. R. Soc. Lond. B. Biol. Sci. 368, 20120289. 10.1098/rstb.2012.0289 23209174 PMC3538459

[B84] Gilbert S. F. (2000). Developmental biology. Sinauer Associates.

[B85] Gilbert S. F. Bosch T. C. G. Ledón-Rettig C. (2015). Eco-Evo-Devo: developmental symbiosis and developmental plasticity as evolutionary agents. Nat. Rev. Genet. 16, 611–622. 10.1038/nrg3982 26370902

[B86] Gilbert S. F. Epel D. (2009). Ecological developmental biology: integrating epigenetics, medicine, and evolution. Sunderland, MA: Sinauer Associates Inc.

[B87] Gissis S. Jablonka E. (2011). Transformations of Lamarckism: from subtle fluids to molecular biology. Cambridge, Massachusetts, USA: MIT Press. 10.7551/mitpress/9780262015141.001.0001

[B88] Gockel J. Robinson S. J. W. Kennington W. J. Goldstein D. B. Partridge L. (2002). Quantitative genetic analysis of natural variation in body size in *Drosophila melanogaster* . Heredity (Edinb). 89, 145–153. 10.1038/sj.hdy.6800121 12136418

[B89] Gomez-Mestre I. Buchholz D. R. (2006). Developmental plasticity mirrors differences among taxa in spadefoot toads linking plasticity and diversity. Proc. Natl. Acad. Sci. U. S. A. 103, 19021–19026. 10.1073/pnas.0603562103 17135355 PMC1748170

[B90] Gordon D. M. (2016). From division of labor to the collective behavior of social insects. Behav. Ecol. Sociobiol. 70, 1101–1108. 10.1007/s00265-015-2045-3 27397966 PMC4917577

[B91] Gotthard K. Nylin S. S. (1995). Adaptive plasticity and plasticity as an adaptation: a selective review of plasticity in animal morphology and life history. Oikos 74, 3. 10.2307/3545669

[B92] Green J. W. M. Stastna J. J. Orbidans H. E. Harvey S. C. (2014). Highly polygenic variation in environmental perception determines dauer larvae formation in growing populations of *Caenorhabditis elegans* . PLoS One 9, e112830. 10.1371/journal.pone.0112830 25393108 PMC4231163

[B93] Greenwood A. K. Jones F. C. Chan Y. F. Brady S. D. Absher D. M. Grimwood J. (2011). The genetic basis of divergent pigment patterns in juvenile threespine sticklebacks. Heredity (Edinb). 107, 155–166. 10.1038/hdy.2011.1 21304547 PMC3136628

[B94] Grishkevich V. Ben-Elazar S. Hashimshony T. Schott D. H. Hunter C. P. Yanai I. (2012). A genomic bias for genotype-environment interactions in *C. elegans* . Mol. Syst. Biol. 8, 587. 10.1038/msb.2012.19 22669615 PMC3397417

[B95] Grishkevich V. Yanai I. (2013). The genomic determinants of genotype × environment interactions in gene expression. Trends Genet. 29, 479–487. 10.1016/j.tig.2013.05.006 23769209

[B96] Gurganus M. C. Fry J. D. Nuzhdin S. V. Pasyukova E. G. Lyman R. F. Mackay T. F. (1998). Genotype-environment interaction at quantitative trait loci affecting sensory bristle number in *Drosophila melanogaster* . Genetics 149, 1883–1898. 9691044 10.1093/genetics/149.4.1883 PMC1460274

[B97] Gutteling E. W. Riksen J. A. G. Bakker J. Kammenga J. E. (2007). Mapping phenotypic plasticity and genotype-environment interactions affecting life-history traits in *Caenorhabditis elegans* . Heredity (Edinb). 98, 28–37. 10.1038/sj.hdy.6800894 16955112

[B98] Guzzo M. M. Mochnacz N. J. Durhack T. Kissinger B. C. Killen S. S. Treberg J. R. (2019). Effects of repeated daily acute heat challenge on the growth and metabolism of a cold-water stenothermal fish. J. Exp. Biol. 222 (Pt 12). 10.1242/jeb.198143 31097605

[B99] Healy T. M. Schulte P. M. (2019). Patterns of alternative splicing in response to cold acclimation in fish. J. Exp. Biol. 222 (Pt 5). 10.1242/jeb.193516 30692167

[B100] Heckwolf M. J. Meyer B. S. Döring T. Eizaguirre C. Reusch T. B. H. (2018). Transgenerational plasticity and selection shape the adaptive potential of sticklebacks to salinity change. Evol. Appl. 11, 1873–1885. 10.1111/eva.12688 30459835 PMC6231470

[B101] Herman J. J. Spencer H. G. Donohue K. Sultan S. E. (2014). How stable ‘should’epigenetic modifications be? Insights from adaptive plasticity and bet hedging. Evolution (N. Y.) 68, 632–643. 10.1111/evo.12324 24274594

[B102] Hosseini S. Brenig B. Tetens J. Sharifi A. R. (2018). Phenotypic plasticity induced using high ambient temperature during embryogenesis in domesticated zebrafish. Danio rerio. Reprod. Domest. Anim. 10.1111/rda.13382 PMC7379563 30472784

[B103] Hoverman J. T. Relyea R. A. (2009). Survival trade-offs associated with inducible defences in snails: the roles of multiple predators and developmental plasticity. Funct. Ecol. 23, 1179–1188. 10.1111/j.1365-2435.2009.01586.x

[B104] International Aphid Genomics Consortium (2010). Genome sequence of the pea aphid *Acyrthosiphon pisum* . PLoS Biol. 8, e1000313. 10.1371/journal.pbio.1000313 20186266 PMC2826372

[B105] Jablonka E. Lamb J. Zeligowski A. (2014). Evolution in four dimensions: genetic, epigenetic, behavioral, and symbolic variation in the history of life. Cambridge, Massachusetts, USA: MIT Press.

[B106] Jakšić A. M. Schlötterer C. (2016). The interplay of temperature and genotype on patterns of alternative splicing in *Drosophila melanogaster* . Genetics 204, 315–325. 10.1534/genetics.116.192310 27440867 PMC5012396

[B107] Jeanson R. Weidenmüller A. (2014). Interindividual variability in social insects - proximate causes and ultimate consequences. Biol. Rev. 89, 671–687. 10.1111/brv.12074 24341677

[B108] Jensen M. P. Allen C. D. Eguchi T. Bell I. P. LaCasella E. L. Hilton W. A. (2018). Environmental warming and feminization of one of the largest sea turtle populations in the world. Curr. Biol. 28, 154-159.e4. 10.1016/j.cub.2017.11.057 29316410

[B109] Kalay G. Lusk R. Dome M. Hens K. Deplancke B. Wittkopp P. J. (2016). Potential direct regulators of the *Drosophila* yellow gene identified by yeast one-hybrid and RNAi screens. G3 (Bethesda). 6, 3419–3430. 10.1534/g3.116.032607 27527791 PMC5068961

[B110] Kamakura M. (2011). Royalactin induces queen differentiation in honeybees. Nature 473, 478–483. 10.1038/nature10093 21516106

[B111] Kelly M. (2019). Adaptation to climate change through genetic accommodation and assimilation of plastic phenotypes. Philos. Trans. R. Soc. B Biol. Sci. 374, 20180176. 10.1098/rstb.2018.0176 PMC6365860 30966963

[B112] Kijimoto T. Snell-Rood E. C. Pespeni M. H. Rocha G. Kafadar K. Moczek A. P. (2014). The nutritionally responsive transcriptome of the polyphenic beetle *Onthophagus taurus* and the importance of sexual dimorphism and body region. Proc. R. Soc. B. Biol. Sci. 281, 20142084–20142084. 10.1098/rspb.2014.2084 PMC4240995 25377458

[B113] Kingsolver J. G. Buckley L. B. (2017). Evolution of plasticity and adaptive responses to climate change along climate gradients. Proc. R. Soc. B. Biol. Sci. 284, 20170386. 10.1098/rspb.2017.0386 PMC5563792 28814652

[B114] Kivelä S. M. Välimäki P. Gotthard K. (2013). Seasonality maintains alternative life-history phenotypes. Evolution (N. Y.) 67, 3145–3160. 10.1111/evo.12181 24151999

[B115] Klingenberg C. P. (2019). Phenotypic plasticity, developmental instability, and robustness: the concepts and how they are connected. Front. Ecol. Evol. 7, 56. 10.3389/fevo.2019.00056

[B116] Koyama T. Mendes C. C. Mirth C. K. (2013). Mechanisms regulating nutrition-dependent developmental plasticity through organ-specific effects in insects. Front. Physiol. 4, 263. 10.3389/fphys.2013.00263 24133450 PMC3783933

[B117] Koyama T. Mirth C. K. Yagi Y. Nishida Y. Kataoka H. O’Connor M. (2016). Growth-blocking peptides as nutrition-sensitive signals for insulin secretion and body size regulation. PLOS Biol. 14, e1002392. 10.1371/journal.pbio.1002392 26928023 PMC4771208

[B118] Kucharski R. Maleszka J. Foret S. Maleszka R. (2008). Nutritional control of reproductive status in honeybees *via* DNA methylation. Science 319, 1827–1830. 10.1126/science.1153069 18339900

[B119] Kulkarni S. S. Denver R. J. Gomez-Mestre I. Buchholz D. R. (2017). Genetic accommodation *via* modified endocrine signalling explains phenotypic divergence among spadefoot toad species. Nat. Commun. 8, 993. 10.1038/s41467-017-00996-5 29051478 PMC5648835

[B120] Lafuente E. Duneau D. Beldade P. (2018). Genetic basis of thermal plasticity variation in *Drosophila melanogaster* body size. PLOS Genet. 14, e1007686. 10.1371/journal.pgen.1007686 30256798 PMC6175520

[B121] Lahiri K. Vallone D. Gondi S. B. Santoriello C. Dickmeis T. Foulkes N. S. (2005). Temperature regulates transcription in the zebrafish circadian clock. PLoS Biol. 3, e351. 10.1371/journal.pbio.0030351 16176122 PMC1233578

[B122] Laland K. N. (2015). On evolutionary causes and evolutionary processes. Behav. Processes 117, 97–104. 10.1016/j.beproc.2014.05.008 24932898

[B123] Laland K. N. Uller T. Feldman M. W. Sterelny K. Müller G. B. Moczek A. (2015). The extended evolutionary synthesis: its structure, assumptions and predictions. Proc. R. Soc. B Biol. Sci. 282, 20151019. 10.1098/rspb.2015.1019 PMC4632619 26246559

[B124] Laland K. Uller T. Feldman M. Sterelny K. Müller G. B. Moczek A. (2014). Does evolutionary theory need a rethink? Nature 514, 161–164. 10.1038/514161a 25297418

[B125] Lande R. (2009). Adaptation to an extraordinary environment by evolution of phenotypic plasticity and genetic assimilation. J. Evol. Biol. 22, 1435–1446. 10.1111/j.1420-9101.2009.01754.x 19467134

[B126] Lande R. (2014). Evolution of phenotypic plasticity and environmental tolerance of a labile quantitative character in a fluctuating environment. J. Evol. Biol. 27, 866–875. 10.1111/jeb.12360 24724972

[B127] Lande R. (2015). Evolution of phenotypic plasticity in colonizing species. Mol. Ecol. 24, 2038–2045. 10.1111/mec.13037 25558898

[B128] Langerhans R. B. Dewitt T. J. (2002). Plasticity constrained: over-generalized induction cues cause maladaptive phenotypes. Evol. Ecol. Res., 4, 857–870.

[B129] Lardies M. (2008). Genetic variation for plasticity in physiological and life-history traits among populations of an invasive species, the terrestrial isopod *Porcellio laevis* . Evol. Ecol. Res. 10, 747–762.

[B130] Ledón-Rettig C. C. Pfennig D. W. Crespi E. J. (2010). Diet and hormonal manipulation reveal cryptic genetic variation: implications for the evolution of novel feeding strategies. Proc. Biol. Sci. 277, 3569–3578. 10.1098/rspb.2010.0877 20573627 PMC2982244

[B131] Ledon-Rettig C. C. Pfennig D. W. Nascone-Yoder N. (2008). Ancestral variation and the potential for genetic accommodation in larval amphibians: implications for the evolution of novel feeding strategies. Evol. Dev. 10, 316–325. 10.1111/j.1525-142X.2008.00240.x 18460093

[B132] Lee K.-Y. Horodyski F. M. (2006). Effects of starvation and mating on corpora allata activity and allatotropin (Manse-AT) gene expression in *Manduca sexta* . Peptides 27, 567–574. 10.1016/j.peptides.2005.08.024 16488512

[B133] Leimar O. Hammerstein P. Van Dooren T. J. M. (2006). A new perspective on developmental plasticity and the principles of adaptive morph determination. Am. Nat. 167, 367–376. 10.1086/499566 16673345

[B134] Levine M. T. Eckert M. L. Begun D. J. (2011). Whole-genome expression plasticity across tropical and temperate *Drosophila melanogaster* populations from Eastern Australia. Mol. Biol. Evol. 28, 249–256. 10.1093/molbev/msq197 20671040 PMC3002243

[B135] Levis N. A. Isdaner A. J. Pfennig D. W. (2018). Morphological novelty emerges from pre-existing phenotypic plasticity. Nat. Ecol. Evol. 2, 1289–1297. 10.1038/s41559-018-0601-8 29988161

[B136] Levis N. A. Pfennig D. W. (2016). Evaluating “plasticity-first” evolution in nature: key criteria and empirical approaches. Trends Ecol. Evol. 31, 563–574. 10.1016/j.tree.2016.03.012 27067134

[B137] Levis N. A. Pfennig D. W. (2018). Phenotypic plasticity, canalization, and the origins of novelty: evidence and mechanisms from amphibians. Semin. Cell Dev. Biol. 88, 80–90. 10.1016/j.semcdb.2018.01.012 29408711

[B138] Levis N. A. Pfennig D. W. (2019). Plasticity-led evolution: evaluating the key prediction of frequency-dependent adaptation. Proc. R. Soc. B. Biol. Sci. 286, 20182754. 10.1098/rspb.2018.2754 PMC6408876 30963848

[B139] Li Y. Álvarez O. A. Gutteling E. W. Tijsterman M. Fu J. Riksen J. A. G. (2006). Mapping determinants of gene expression plasticity by genetical genomics in *C. elegans* . PLoS Genet. 2, e222. 10.1371/journal.pgen.0020222 17196041 PMC1756913

[B140] Ludewig A. H. Schroeder F. C. (2013). Ascaroside signaling in *C. elegans* . WormBook 18, 1–22. 10.1895/wormbook.1.155.1 PMC3758900 23355522

[B141] Lyko F. Foret S. Kucharski R. Wolf S. Falckenhayn C. Maleszka R. (2010). The honey bee epigenomes: differential methylation of brain DNA in queens and workers. PLoS Biol. 8, e1000506. 10.1371/journal.pbio.1000506 21072239 PMC2970541

[B142] Maleszka R. (2008). Epigenetic integration of environmental and genomic signals in honey bees: the critical interplay of nutritional, brain and reproductive networks. Epigenetics 3, 188–192. 10.4161/epi.3.4.6697 18719401

[B143] Marinković M. de Leeuw W. C. de Jong M. Kraak M. H. S. Admiraal W. Breit T. M. (2012). Combining next-generation sequencing and microarray technology into a transcriptomics approach for the non-model organism *Chironomus riparius* . PLoS One 7, e48096. 10.1371/journal.pone.0048096 23133553 PMC3485019

[B144] Martin A. Orgogozo V. (2013). The loci of repeated evolution: a catalog of genetic hotspots of phenotypic variation. Evolution (N. Y.) 67, 1235–1250. 10.1111/evo.12081 23617905

[B145] Martins N. E. Faria V. G. Nolte V. Schlotterer C. Teixeira L. Sucena E. (2014). Host adaptation to viruses relies on few genes with different cross-resistance properties. Proc. Natl. Acad. Sci. 111, 5938–5943. 10.1073/pnas.1400378111 24711428 PMC4000853

[B146] Mateus A. R. A. Marques-Pita M. Oostra V. Lafuente E. Brakefield P. M. Zwaan B. J. (2014). Adaptive developmental plasticity: compartmentalized responses to environmental cues and to corresponding internal signals provide phenotypic flexibility. BMC Biol. 12, 97. 10.1186/s12915-014-0097-x 25413287 PMC4275937

[B147] Matsui T. Ehrenreich I. M. (2016). Gene-environment interactions in stress response contribute additively to a genotype-environment interaction. PLOS Genet. 12, e1006158. 10.1371/journal.pgen.1006158 27437938 PMC4954657

[B148] McGuigan K. Nishimura N. Currey M. Hurwit D. Cresko W. A. (2011). Cryptic genetic variation and body size evolution in threespine stickleback. Evolution (N. Y.) 65, 1203–1211. 10.1111/j.1558-5646.2010.01195.x 21463296

[B149] Mendes C. C. Mirth C. K. (2016). Stage-specific plasticity in ovary size is regulated by insulin/insulin-like growth factor and ecdysone signaling in *Drosophila* . Genetics 202, 703–719. 10.1534/genetics.115.179960 26715667 PMC4788244

[B150] Merchant-Larios H. Díaz-Hernández V. (2013). Environmental sex determination mechanisms in reptiles. Sex. Dev. 7, 95–103. 10.1159/000341936 22948613

[B151] Merilä J. Hendry A. P. (2014). Climate change, adaptation, and phenotypic plasticity: the problem and the evidence. Evol. Appl. 7, 1–14. 10.1111/eva.12137 24454544 PMC3894893

[B152] Mesoudi A. Blanchet S. Charmantier A. Danchin É. Fogarty L. Jablonka E. (2013). Is non-genetic inheritance just a proximate mechanism? A corroboration of the extended evolutionary synthesis. Biol. Theory 7, 189–195. 10.1007/s13752-013-0091-5

[B153] Mills L. S. Zimova M. Oyler J. Running S. Abatzoglou J. T. Lukacs P. M. (2013). Camouflage mismatch in seasonal coat color due to decreased snow duration. Proc. Natl. Acad. Sci. 110, 7360–7365. 10.1073/pnas.1222724110 23589881 PMC3645584

[B154] Mirth C. K. Shingleton A. W. (2012). Integrating body and organ size in *Drosophila*: recent advances and outstanding problems. Front. Endocrinol. (Lausanne). 3, 49. 10.3389/fendo.2012.00049 22654869 PMC3356080

[B155] Mirth C. K. Shingleton A. W. (2014). The roles of juvenile hormone, insulin/target of rapamycin, and ecydsone signaling in regulating body size in *Drosophila* . Commun. Integr. Biol. 7, e971568. 10.4161/cib.29240 PMC4594587 26842847

[B156] Mirth C. K. Tang H. Y. Makohon-Moore S. C. Salhadar S. Gokhale R. H. Warner R. D. (2014). Juvenile hormone regulates body size and perturbs insulin signaling in *Drosophila* . Proc. Natl. Acad. Sci. 111, 7018–7023. 10.1073/pnas.1313058111 24778227 PMC4024895

[B157] Mirth C. K. Truman W. J. Riddiford M. L. (2009). The Ecdysone receptor controls the post-critical weight switch to nutrition-independent differentiation in *Drosophila* wing imaginal discs. Development 136, 2345–2353. 10.1242/dev.032672 19515698 PMC2729347

[B158] Mitaka Y. Kobayashi K. Mikheyev A. Tin M. M. Y. Watanabe Y. Matsuura K. (2016). Caste-specific and sex-specific expression of chemoreceptor genes in a termite. PLoS One 11, e0146125. 10.1371/journal.pone.0146125 26760975 PMC4712011

[B159] Mitchell T. S. Janzen F. J. Warner D. A. (2018). Quantifying the effects of embryonic phenotypic plasticity on adult phenotypes in reptiles: a review of current knowledge and major gaps. J. Exp. Zool. Pt. A Ecol. Integr. Physiol. 329 (4–5), 203–214. 10.1002/jez.2187 29869377

[B160] Miyakawa H. Imai M. Sugimoto N. Ishikawa Y. Ishikawa A. Ishigaki H. (2010). Gene up-regulation in response to predator kairomones in the water flea, *Daphnia pulex* . BMC Dev. Biol. 10, 45. 10.1186/1471-213X-10-45 20433737 PMC2888767

[B161] Moczek A. (1998). Horn polyphenism in the beetle *Onthophagus taurus*: larval diet quality and plasticity in parental investment determine adult body size and male horn morphology. Behav. Ecol. 9, 636–641. 10.1093/beheco/9.6.636

[B162] Moczek A. P. (2002). Allometric plasticity in a polyphenic beetle. Ecol. Entomol. 27, 58–67. 10.1046/j.0307-6946.2001.00385.x

[B163] Moczek A. P. Sultan S. Foster S. Ledón-Rettig C. Dworkin I. Nijhout H. F. (2011). The role of developmental plasticity in evolutionary innovation. Proc. R. Soc. B. Biol. Sci. 278, 2705–2713. 10.1098/rspb.2011.0971 PMC3145196 21676977

[B164] Monteiro A. (2015). Origin, development, and evolution of butterfly eyespots. Annu. Rev. Entomol. 60, 253–271. 10.1146/annurev-ento-010814-020942 25341098

[B165] Monteiro A. Tong X. Bear A. Liew S. F. Bhardwaj S. Wasik B. R. (2015). Differential expression of Ecdysone receptor leads to variation in phenotypic plasticity across serial homologs. PLOS Genet. 11, e1005529. 10.1371/journal.pgen.1005529 26405828 PMC4583414

[B166] Murren C. J. Auld J. R. Callahan H. Ghalambor C. K. Handelsman C. A. Heskel M. A. (2015). Constraints on the evolution of phenotypic plasticity: limits and costs of phenotype and plasticity. Heredity (Edinb). 115, 293–301. 10.1038/hdy.2015.8 25690179 PMC4815460

[B167] Muschick M. Barluenga M. Salzburger W. Meyer A. (2011). Adaptive phenotypic plasticity in the *Midas cichlid* fish pharyngeal jaw and its relevance in adaptive radiation. BMC Evol. Biol. 11, 116. 10.1186/1471-2148-11-116 21529367 PMC3103464

[B168] Newman S. A. Müller G. B. (2000). Epigenetic mechanisms of character origination. J. Exp. Zool. 288, 304–317. 10.1002/1097-010X(20001215)288:4<304::AID-JEZ3>3.0.CO;2-G 11144279

[B169] Nicoglou A. (2015). The evolution of phenotypic plasticity: genealogy of a debate in genetics. Stud. Hist. Philos. Sci. Pt. C Stud. Hist. Philos. Biol. Biomed. Sci. 50, 67–76. 10.1016/j.shpsc.2015.01.003 25636689

[B170] Nijhout F. H. (2003a). Development and evolution of adaptive polyphenisms. Evol. Dev. 5, 9–18. 10.1046/j.1525-142X.2003.03003.x 12492404

[B171] Nijhout H. F. (2003b). The control of body size in insects. Dev. Biol. 261, 1–9. 10.1016/S0012-1606(03)00276-8 12941617

[B172] Nijhout H. F. Sadre-Marandi F. Best J. Reed M. C. (2017). Systems biology of phenotypic robustness and plasticity. Integr. Comp. Biol. 57, 171–184. 10.1093/icb/icx076 28859407

[B173] Noble D. W. A. Stenhouse V. Riley J. L. Warner D. A. While G. M. Du W.-G. (2018). A comprehensive database of thermal developmental plasticity in reptiles. Sci. Data 5, 180138. 10.1038/sdata.2018.138 30015809 PMC6049033

[B174] Nussey D. H. Postma E. Gienapp P. Visser M. E. (2005). Selection on heritable phenotypic plasticity in a wild bird population. Science 310, 304–306. 10.1126/science.1117004 16224020

[B175] Oomen R. A. Hutchings J. A. (2017). Transcriptomic responses to environmental change in fishes: insights from RNA sequencing. FACETS 2, 610–641. 10.1139/facets-2017-0015

[B176] Oostra V. Mateus A. R. A. van der Burg K. R. L. Piessens T. van Eijk M. Brakefield P. M. (2014). Ecdysteroid hormones link the juvenile environment to alternative adult life histories in a seasonal insect. Am. Nat. 184, E79–E92. 10.1086/677260 25141151

[B177] Oostra V. Saastamoinen M. Zwaan B. J. Wheat C. W. (2018). Strong phenotypic plasticity limits potential for evolutionary responses to climate change. Nat. Commun. 9, 1005. 10.1038/s41467-018-03384-9 29520061 PMC5843647

[B178] Ørsted M. Rohde P. D. Hoffmann A. A. Sørensen P. Kristensen T. N. (2018). Environmental variation partitioned into separate heritable components. Evolution (N. Y.) 72, 136–152. 10.1111/evo.13391 29125643

[B179] Oufiero C. E. Whitlow K. R. (2016). The evolution of phenotypic plasticity in fish swimming. Curr. Zool. 62, 475–488. 10.1093/cz/zow084 29491937 PMC5804253

[B180] Paaby A. B. Rockman M. V. (2014). Cryptic genetic variation: evolution’s hidden substrate. Nat. Rev. Genet. 15, 247–258. 10.1038/nrg3688 24614309 PMC4737706

[B181] Pardo-Diaz C. Salazar C. Jiggins C. D. (2015). Towards the identification of the loci of adaptive evolution. Methods Ecol. Evol. 6, 445–464. 10.1111/2041-210X.12324 25937885 PMC4409029

[B182] Pener M. P. Simpson S. J. (2009). Locust phase polyphenism: an update. Adv. In Insect Phys. 36, 1–272. 10.1016/S0065-2806(08)36001-9

[B183] Peng I.-F. Berke B. A. Zhu Y. Lee W.-H. Chen W. Wu C.-F. (2007). Temperature-dependent developmental plasticity of *Drosophila* neurons: cell-autonomous roles of membrane excitability, Ca^2+^ influx, and cAMP signaling. J. Neurosci. 27, 12611–12622. 10.1523/JNEUROSCI.2179-07.2007 18003840 PMC6673343

[B184] Pennisi E. (2018). Buying time. Science 362, 988–991. 10.1126/science.362.6418.988 30498109

[B185] Pfennig D. W. (1992). Polyphenism in spadefoot toad tadpoles as a locally-adjusted evolutionarily stable strategy. Evolution (N. Y.) 46, 1408–1420. 10.1111/j.1558-5646.1992.tb01133.x 28568981

[B186] Pfennig D. W. Wund M. A. Snell-Rood E. C. Cruickshank T. Schlichting C. D. Moczek A. P. (2010). Phenotypic plasticity’s impacts on diversification and speciation. Trends Ecol. Evol. 25, 459–467. 10.1016/j.tree.2010.05.006 20557976

[B187] Piggott J. J. Townsend C. R. Matthaei C. D. (2015). Reconceptualizing synergism and antagonism among multiple stressors. Ecol. Evol. 5, 1538–1547. 10.1002/ece3.1465 25897392 PMC4395182

[B188] Pigliucci M. (2005). Evolution of phenotypic plasticity: where are we going now? Trends Ecol. Evol. 20, 481–486. 10.1016/j.tree.2005.06.001 16701424

[B189] Pigliucci M. (2007). Do we need an extended evolutionary synthesis? Evolution (N. Y.) 61, 2743–2749. 10.1111/j.1558-5646.2007.00246.x 17924956

[B190] Pigliucci M. (2009). An extended synthesis for evolutionary biology. Ann. N. Y. Acad. Sci. 1168, 218–228. 10.1111/j.1749-6632.2009.04578.x 19566710

[B191] Projecto-Garcia J. Biddle J. F. Ragsdale E. J. (2017). Decoding the architecture and origins of mechanisms for developmental polyphenism. Curr. Opin. Genet. Dev. 47, 1–8. 10.1016/j.gde.2017.07.015 28810163

[B192] Promislow D. (2005). A regulatory network analysis of phenotypic plasticity in yeast. Am. Nat. 165, 515–523. 10.1086/429161 15795849

[B193] Przybylo R. Sheldon B. C. Merila J. (2000). Climatic effects on breeding and morphology: evidence for phenotypic plasticity. J. Anim. Ecol. 69, 395–403. 10.1046/j.1365-2656.2000.00401.x

[B194] Queitsch C. Sangster T. A. Lindquist S. (2002). Hsp90 as a capacitor of phenotypic variation. Nature 417, 618–624. 10.1038/nature749 12050657

[B195] Ragsdale E. J. Ivers N. A. (2016). Specialization of a polyphenism switch gene following serial duplications in *Pristionchus* nematodes. Evolution (N. Y.) 70, 2155–2166. 10.1111/evo.13011 27436344

[B196] Ragsdale E. J. Müller M. R. Rödelsperger C. Sommer R. J. (2013). A developmental switch coupled to the evolution of plasticity acts through a sulfatase. Cell 155, 922–933. 10.1016/j.cell.2013.09.054 24209628

[B197] Réale D. McAdam A. G. Boutin S. Berteaux D. (2003). Genetic and plastic responses of a northern mammal to climate change. Proc. Biol. Sci. 270, 591–596. 10.1098/rspb.2002.2224 12769458 PMC1691280

[B198] Reed T. E. Waples R. S. Schindler D. E. Hard J. J. Kinnison M. T. (2010). Phenotypic plasticity and population viability: the importance of environmental predictability. Proc. Biol. Sci. 277, 3391–3400. 10.1098/rspb.2010.0771 20554553 PMC2982227

[B199] Rocha F. Medeiros H. F. Klaczko L. B. (2009). The reaction norm for abdominal pigmentation and its curve in *Drosophila mediopunctata* depend on the mean phenotypic value. Evolution (N. Y.) 63, 280–287. 10.1111/j.1558-5646.2008.00503.x 18752606

[B200] Rodrigues Y. K. van Bergen E. Alves F. Duneau D. Beldade P. (2017). Complex effects of day and night temperature fluctuations on thermally plastic traits in an experimental model of adaptive seasonal plasticity. BioRxiv. 10.1101/207258 34097756

[B201] Runcie D. E. Garfield D. A. Babbitt C. C. Wygoda J. A. Mukherjee S. Wray G. A. (2012). Genetics of gene expression responses to temperature stress in a sea urchin gene network. Mol. Ecol. 21, 4547–4562. 10.1111/j.1365-294X.2012.05717.x 22856327 PMC3866972

[B202] Russell J. J. Theriot J. A. Sood P. Marshall W. F. Landweber L. F. Fritz-Laylin L. (2017). Non-model model organisms. BMC Biol. 15, 55. 10.1186/s12915-017-0391-5 28662661 PMC5492503

[B203] Saastamoinen M. Brommer J. E. Brakefield P. M. Zwaan B. J. (2013). Quantitative genetic analysis of responses to larval food limitation in a polyphenic butterfly indicates environment- and trait-specific effects. Ecol. Evol. 3, 3576–89. 10.1002/ece3.718 PMC3797501 24223292

[B204] Saltz J. B. Bell A. M. Flint J. Gomulkiewicz R. Hughes K. A. Keagy J. (2018). Why does the magnitude of genotype-by-environment interaction vary? Ecol. Evol. 10.1002/ece3.4128 PMC6024136 29988442

[B205] Sangster T. A. Lindquist S. Queitsch C. (2004). Under cover: causes, effects and implications of Hsp90-mediated genetic capacitance. BioEssays 26, 348–362. 10.1002/bies.20020 15057933

[B206] Santure A. W. Garant D. (2018). Wild GWAS-association mapping in natural populations. Mol. Ecol. Resour. 18, 729–738. 10.1111/1755-0998.12901 29782705

[B207] Sasabe M. Takami Y. Sota T. (2007). The genetic basis of interspecific differences in genital morphology of closely related carabid beetles. Heredity (Edinb). 98, 385–391. 10.1038/sj.hdy.6800952 17327872

[B208] Savolainen O. Lascoux M. Merilä J. (2013). Ecological genomics of local adaptation. Nat. Rev. Genet. 14, 807–820. 10.1038/nrg3522 24136507

[B209] Saxon A. D. O’Brien E. K. Bridle J. R. (2018). Temperature fluctuations during development reduce male fitness and may limit adaptive potential in tropical rainforest *Drosophila* . J. Evol. Biol. 31, 405–415. 10.1111/jeb.13231 29282784

[B210] Scheiner S. M. Lyman R. F. (1989). The genetics of phenotypic plasticity I. Heritability. J. Evol. Biol. 2, 95–107. 10.1046/j.1420-9101.1989.2020095.x

[B211] Scheiner S. M. Lyman R. F. (1991). The genetics of phenotypic plasticity. II. Response to selection. J. Evol. Biol. 4, 23–50. 10.1046/j.1420-9101.1991.4010023.x

[B212] Schlichting C. D. Pigliucci M. (1993). Control of phenotypic plasticity *via* regulatory genes. Am. Nat. 142, 366–370. 10.1086/285543 19425982

[B213] Schlichting C. D. Wund M. A. (2014). Phenotypic plasticity and epigenetic marking: an assessment of evidence for genetic accommodation. Evolution (N. Y.) 68, 656–672. 10.1111/evo.12348 24410266

[B214] Schlichting C. Pigliucci M., (1998). Phenotypic evolution: a reaction norm perspective. Sunderland, Massachusetts: Sinauer Associates Inc.

[B215] Schneider R. F. Meyer A. (2017). How plasticity, genetic assimilation and cryptic genetic variation may contribute to adaptive radiations. Mol. Ecol. 26, 330–350. 10.1111/mec.13880 27747962

[B216] Schulz N. K. E. Sell M. P. Ferro K. Kleinhölting N. Kurtz J. (2019). Transgenerational developmental effects of immune priming in the red flour beetle tribolium castaneum. Front. Physiol. 10, 98. 10.3389/fphys.2019.00098 30837885 PMC6389831

[B217] Sentis A. Bertram R. Dardenne N. Ramon-Portugal F. Espinasse G. Louit I. (2018). Evolution without standing genetic variation: change in transgenerational plastic response under persistent predation pressure. Heredity (Edinb). 10.1038/s41437-018-0108-8 PMC6082885 29959428

[B218] Sgrò C. M. Terblanche J. S. Hoffmann A. A. (2016). What can plasticity contribute to insect responses to climate change? Annu. Rev. Entomol. 61, 433–451. 10.1146/annurev-ento-010715-023859 26667379

[B219] Shoemaker-Daly C. M. Jackson K. Yatsu R. Matsumoto Y. Crews D. (2010). Genetic network underlying temperature-dependent sex determination is endogenously regulated by temperature in isolated cultured *Trachemys scripta* gonads. Dev. Dyn. 239, 1061–1075. 10.1002/dvdy.22266 20235200

[B220] Sikkink K. L. Reynolds R. M. Ituarte C. M. Cresko W. A. Phillips P. C. (2014). Rapid evolution of phenotypic plasticity and shifting thresholds of genetic assimilation in the nematode *Caenorhabditis remanei* . G3 (Bethesda). 4, 1103–1112. 10.1534/g3.114.010553 24727288 PMC4065253

[B221] Silvestre F. Gillardin V. Dorts J. (2012). Proteomics to assess the role of phenotypic plasticity in aquatic organisms exposed to pollution and global warming. Integr. Comp. Biol. 52, 681–694. 10.1093/icb/ics087 22641836

[B222] Simola D. F. Graham R. J. Brady C. M. Enzmann B. L. Desplan C. Ray A. (2016). Epigenetic (re)programming of caste-specific behavior in the ant *Camponotus floridanus* . Science 351, aac6633–aac6633. 10.1126/science.aac6633 PMC5057185 26722000

[B223] Simola D. F. Wissler L. Donahue G. Waterhouse R. M. Helmkampf M. Roux J. (2013a). Social insect genomes exhibit dramatic evolution in gene composition and regulation while preserving regulatory features linked to sociality. Genome Res. 23, 1235–1247. 10.1101/gr.155408.113 23636946 PMC3730098

[B224] Simola D. F. Ye C. Mutti N. S. Dolezal K. Bonasio R. Liebig J. (2013b). A chromatin link to caste identity in the carpenter ant *Camponotus floridanus* . Genome Res. 23, 486–496. 10.1101/gr.148361.112 23212948 PMC3589537

[B225] Simons A. M. (2011). Modes of response to environmental change and the elusive empirical evidence for bet hedging. Proc. R. Soc. B. Biol. Sci. 278, 1601–1609. 10.1098/rspb.2011.0176 PMC3081777 21411456

[B226] Simpson S. J. Sword G. A. Lo N. (2011). Polyphenism in insects. Curr. Biol. 21, R738–R749. 10.1016/j.cub.2011.06.006 21959164

[B227] Smith C. R. Anderson K. E. Tillberg C. V. Gadau J. Suarez A. V. (2008). Caste determination in a polymorphic social insect: nutritional, social, and genetic factors. Am. Nat. 172, 497–507. 10.1086/590961 18707530

[B228] Smith G. Ritchie M. G. (2013). How might epigenetics contribute to ecological speciation? Curr. Zool. 59, 686–696. 10.1093/czoolo/59.5.686

[B229] Snell-Rood E. C. (2012). Selective processes in development: implications for the costs and benefits of phenotypic plasticity. Integr. Comp. Biol. 52, 31–42. 10.1093/icb/ics067 22544286

[B230] Snell-Rood E. C. Kobiela M. E. Sikkink K. L. Shephard A. M. (2018). Mechanisms of plastic rescue in novel environments. Annu. Rev. Ecol. Evol. Syst. 49, 331–354. 10.1146/annurev-ecolsys-110617-062622

[B231] Snell-Rood E. C. Van Dyken J. D. Cruickshank T. Wade M. J. Moczek A. P. (2010). Toward a population genetic framework of developmental evolution: the costs, limits, and consequences of phenotypic plasticity. BioEssays 32, 71–81. 10.1002/bies.200900132 20020499 PMC3151734

[B232] Song H. Foquet B. Mariño-Pérez R. Woller D. A. (2017). Phylogeny of locusts and grasshoppers reveals complex evolution of density-dependent phenotypic plasticity. Sci. Rep. 7, 6606. 10.1038/s41598-017-07105-y 28747803 PMC5529561

[B233] Standen E. M. Du T. Y. Larsson H. C. E. (2014). Developmental plasticity and the origin of tetrapods. Nature 513, 54–58. 10.1038/nature13708 25162530

[B234] Stern D. L. (2000). Evolutionary developmental biology and the problem of variation. Evolution (N. Y.) 54, 1079–1091. 10.1111/j.0014-3820.2000.tb00544.x 11005278

[B235] Stoehr A. M. Wojan E. M. (2016). Multiple cues influence multiple traits in the phenotypically plastic melanization of the cabbage white butterfly. Oecologia 182, 691–701. 10.1007/s00442-016-3694-2 27417547

[B236] Sultan S. E. (2000). Phenotypic plasticity for plant development, function and life history. Trends Plant Sci. 5, 537–542. 10.1016/S1360-1385(00)01797-0 11120476

[B237] Sultan S. E. (2017). Developmental plasticity: re-conceiving the genotype. Interface Focus 7, 20170009. 10.1098/rsfs.2017.0009 28839928 PMC5566816

[B238] Susoy V. Ragsdale E. J. Kanzaki N. Sommer R. J. (2015). Rapid diversification associated with a macroevolutionary pulse of developmental plasticity. Elife 4, e05463. 10.7554/eLife.05463 PMC4357287 25650739

[B239] Suzuki Y. Nijhout H. F. (2006). Evolution of a polyphenism by genetic accommodation. Science 311, 650–652. 10.1126/science.1118888 16456077

[B240] Svensson E. I. (2018). On reciprocal causation in the evolutionary process. Evol. Biol. 45, 1–14. 10.1007/s11692-017-9431-x 29497217 PMC5816131

[B241] Teplitsky C. Mills J. A. Alho J. S. Yarrall J. W. Merilä J. (2008). Bergmann’s rule and climate change revisited: disentangling environmental and genetic responses in a wild bird population. Proc. Natl. Acad. Sci. U. S. A. 105, 13492–13496. 10.1073/pnas.0800999105 18757740 PMC2533217

[B242] Tétard-Jones C. Kertesz M. A. Preziosi R. F. (2011). Quantitative trait loci mapping of phenotypic plasticity and genotype-environment interactions in plant and insect performance. Philos. Trans. R. Soc. Lond. B. Biol. Sci. 366, 1368–1379. 10.1098/rstb.2010.0356 21444311 PMC3081578

[B243] Thompson J. D. (1991). Phenotypic plasticity as a component of evolutionary change. Trends Ecol. Evol. 6, 246–249. 10.1016/0169-5347(91)90070-E 21232470

[B244] Torres-Dowdall J. Handelsman C. A. Reznick D. N. Ghalambor C. K. (2012). Local adaptation and the evolution of phenotypic plasticity in Trinidadian guppies (*Poecilia reticulata*). Evolution (N. Y.) 66, 3432–3443. 10.1111/j.1558-5646.2012.01694.x 23106708

[B245] True J. R. (2003). Insect melanism: the molecules matter. Trends Ecol. Evol. 18, 640–647. 10.1016/j.tree.2003.09.006

[B246] Tufto J. (2000). The evolution of plasticity and nonplastic spatial and temporal adaptations in the presence of imperfect environmental cues. Am. Nat. 156, 121–130. 10.1086/303381 10856196

[B247] Tufto J. (2015). Genetic evolution, plasticity, and bet-hedging as adaptive responses to temporally autocorrelated fluctuating selection: a quantitative genetic model. Evolution (N. Y.) 69, 2034–2049. 10.1111/evo.12716 26140293

[B248] Turcotte M. M. Levine J. M. (2016). Phenotypic plasticity and species coexistence. Trends Ecol. Evol. 31, 803–813. 10.1016/j.tree.2016.07.013 27527257

[B249] van Bergen E. Beldade P. (2019). Seasonal plasticity in anti-predatory strategies: Matching of color and color preference for effective crypsis. Evol. Lett. 10.1002/evl3.113 PMC6546441 31171986

[B250] van Bergen E. Osbaldeston D. Kodandaramaiah U. Brattström O. Aduse-Poku K. Brakefield P. M. (2017). Conserved patterns of integrated developmental plasticity in a group of polyphenic tropical butterflies. BMC Evol. Biol. 17, 59. 10.1186/s12862-017-0907-1 28241743 PMC5327525

[B251] Van Buskirk J. (2017). Spatially heterogeneous selection in nature favors phenotypic plasticity in anuran larvae. Evolution (N. Y.) 71, 1670–1685. 10.1111/evo.13236 28346658

[B252] Vellichirammal N. N. Gupta P. Hall T. A. Brisson J. A. (2017). Ecdysone signaling underlies the pea aphid transgenerational wing polyphenism. Proc. Natl. Acad. Sci. U. S. A. 114, 1419–1423. 10.1073/pnas.1617640114 28115695 PMC5307454

[B253] Via S. (1993). Adaptive phenotypic plasticity: target or by-product of selection in a variable environment? Am. Nat. 142, 352–365. 10.1086/285542 19425981

[B254] Via S. Lande R. (1985). Genotype-environment interaction and the evolution of phenotypic plasticity. Evolution (N. Y.) 39, 505–522. 10.1111/j.1558-5646.1985.tb00391.x 28561964

[B255] Vieira C. Pasyukova E. G. Zeng Z. B. Hackett J. B. Lyman R. F. Mackay T. F. (2000). Genotype-environment interaction for quantitative trait loci affecting life span in *Drosophila melanogaster* . Genetics 154, 213–227. 10628982 10.1093/genetics/154.1.213 PMC1460900

[B256] Visser M. E. Caro S. P. van Oers K. Schaper S. V. Helm B. (2010). Phenology, seasonal timing and circannual rhythms: towards a unified framework. Philos. Trans. R. Soc. B Biol. Sci. 365, 3113–3127. 10.1098/rstb.2010.0111 PMC2981940 20819807

[B257] Waddington C. H. (1953). Genetic assimilation of an acquired character. Evolution (N. Y.) 7, 118–126. 10.1111/j.1558-5646.1953.tb00070.x

[B258] Walsh M. R. Castoe T. Holmes J. Packer M. Biles K. Walsh M. (2016). Local adaptation in transgenerational responses to predators. Proc. R. Soc. B. Biol. Sci. 283, 20152271. 10.1098/rspb.2015.2271 PMC4795015 26817775

[B259] Warkentin K. M. (2011). Environmentally cued hatching across taxa: embryos respond to risk and opportunity. Integr. Comp. Biol. 51, 14–25. 10.1093/icb/icr017 21705799

[B260] Wellband K. W. Heath D. D. (2017). Plasticity in gene transcription explains the differential performance of two invasive fish species. Evol. Appl. 10, 563–576. 10.1111/eva.12463 28616064 PMC5469171

[B261] West-Eberhard M. J. (2003). Developmental plasticity and evolution. Oxford, UK: Oxford University Press.

[B262] West-Eberhard M. J. (2005). Developmental plasticity and the origin of species differences. Proc. Natl. Acad. Sci. U. S. A. 102 Suppl, 6543–6549. 10.1073/pnas.0501844102 15851679 PMC1131862

[B263] Westerman E. L. Chirathivat N. Schyling E. Monteiro A. (2014). Mate preference for a phenotypically plastic trait is learned, and may facilitate preference-phenotype matching. Evolution (N. Y.) 68, 1661–1670. 10.1111/evo.12381 24528407

[B264] Whitehead A. Crawford D. L. (2006). Variation within and among species in gene expression: raw material for evolution. Mol. Ecol. 15, 1197–1211. 10.1111/j.1365-294X.2006.02868.x 16626448

[B265] Wijngaarden P. J. Brakefield P. M. (2000). The genetic basis of eyespot size in the butterfly *Bicyclus anynana*: an analysis of line crosses. Heredity (Edinb). 85 Pt 5, 471–479. 10.1046/j.1365-2540.2000.00786.x 11122426

[B266] Wund M. A. (2012). Assessing the impacts of phenotypic plasticity on evolution. Integr. Comp. Biol. 52, 5–15. 10.1093/icb/ics050 22544287

[B267] Wund M. A. A. Baker J. A. A. Clancy B. Golub J. L. L. Foster S. A. A. (2008). A test of the “flexible stem” model of evolution: ancestral plasticity, genetic accommodation, and morphological divergence in the threespine stickleback radiation. Am. Nat. 172, 449–462. 10.1086/590966 18729721

[B268] Wurm Y. Wang J. Riba-Grognuz O. Corona M. Nygaard S. Hunt B. G. (2011). The genome of the fire ant *Solenopsis invicta* . Proc. Natl. Acad. Sci. U. S. A. 108, 5679–5684. 10.1073/pnas.1009690108 21282665 PMC3078418

[B269] Xue B. Leibler S. (2018). Benefits of phenotypic plasticity for population growth in varying environments. Proc. Natl. Acad. Sci. 115, 12745–12750. 10.1073/pnas.1813447115 30478048 PMC6294897

[B270] Zhou S. Campbell T. G. Stone E. Mackay T. F. C. Anholt R. R. H. (2012). Phenotypic plasticity of the *Drosophila* transcriptome. PLoS Genet. 8, e1002593. 10.1371/journal.pgen.1002593 22479193 PMC3315458

[B271] Zimova M. Mills L. S. Nowak J. J. (2016). High fitness costs of climate change-induced camouflage mismatch. Ecol. Lett. 19, 299–307. 10.1111/ele.12568 26799459

